# A prognostic risk prediction model for gastric cancer based on the EFNA4 and ETS1 regulatory axis in tumor cells

**DOI:** 10.1038/s41598-025-21728-6

**Published:** 2025-10-29

**Authors:** Yixuan Chen, Ning Wang, Junkun Wang, Shupei Li, Wenchen Zhang, Wenjiang Deng, Jingjing Ye, Zhoujuan Yao, Hui Zhang, Fengsong Wang, Wenbin Wang

**Affiliations:** 1 Department of General Surgery, The First Affiliated Hospital of Anhui Medical University, 100 Huaihai Avenue, Hefei, 230002 Anhui China; 2 School of Life Sciences, Anhui Medical University, 81 Meishan Road, Hefei, 230032 Anhui China; 3 Anhui Public Health Clinical Center, Hefei, 230022 China; 4 Harvard T.H. Chan School of Public Health, Harvard University, 677 Huntington Ave, Boston, MA 02115 USA; 5 Department of General Surgery, The Second Affiliated Hospital of Anhui Medical University, 678 Furong Road, Hefei, 230601 Anhui China

**Keywords:** Gastric cancer (GC), EFNA4, ETS1, Immune infiltration, Signaling pathways, Prognostic model, Data mining, Gene regulatory networks, Genome informatics, Cancer genomics, Cancer genetics, Translational research, Gastroenterology

## Abstract

Gastric cancer (GC) is a major cause of cancer-related deaths worldwide, and is characterised by intricate molecular mechanisms. However, analysis of its molecular and clinical characteristics is complicated by its histological and etiological heterogeneity. Dysregulation of the PI3K-Akt signalling pathway is common in GC. In this study, we have identified the hub gene *Ephrin A4* (*EFNA4*) in the PI3K-Akt pathway based on transcriptome data from The Cancer Genome Atlas (TCGA) and Gene Expression Omnibus (GEO) databases, and *ETS Proto-Oncogene 1* (*ETS1*) was a gene related to EFNA4. Using publicly accessible datasets, we conducted bioinformatics analyses to evaluate the expression profiles, functional roles, and prognostic significance of EFNA4 and ETS1 and further explored their relationship in GC. Subsequently, consensus clustering was performed on 373 TCGA-STAD datasets based on the expression matrices of EFNA4 and ETS1 to assess their interconnections with relevant signalling cascades and immune system components. To address the challenges posed by tumour heterogeneity and reveal the expression patterns of EFNA4 and ETS1 in GC tissues, we performed reanalysis of single-cell RNA sequencing (scRNA-seq) data of GC samples. We constructed a tumour-based risk signature for GC based on EFNA4, ETS1, and marker genes of the tumour cell cluster. The prognostic value of the prognosis prediction model was verified using TCGA database to facilitate the clinical application of tumour cell features in GC prognosis. Our study reveals EFNA4 and ETS1 expression patterns in GC, implicating their roles in pathogenesis. An integrated EFNA4-ETS1 prognostic model improves GC risk stratification. Although EFNA4 has been shown to promote metastasis in liver cancer, the contradictory mechanism of its high expression and good prognosis in GC remains to be elucidated, which may involve its antagonistic effects with ETS1, and requires further exploration.

**Supplementary Information:**

The online version contains supplementary material available at 10.1038/s41598-025-21728-6.

## Introduction

Gastric cancer (GC) is a major global health concern, ranking the fifth most diagnosed cancer and the third leading cause of cancer-related deaths^1^. A significant proportion of patients are diagnosed at an advanced stage, which results in limited treatment effectiveness and poor prognosis^2^. Histological and biomarker analyses that guides early detection and therapeutic decisions are critical for diagnosis, classification, and characterisation. Genetic aberrations, including growth factor/receptor overexpression, alterations in DNA damage response, and genomic instability, contribute to gastric carcinogenesis^3^. Molecular pathology helps in diagnosis and treatment by elucidating cancer-driving pathways^2,4–6^.

The PI3K-AKT pathway has been shown to be essential for normal cellular processes. Genes in the PI3K-AKT pathway are frequently dysregulated in human cancers, including GC, and affect autophagy, epithelial-mesenchymal transition (EMT), apoptosis, chemoresistance, and metastasis in cancer cells^7–9^. Investigating the key genes and molecular interactions involved in this pathway may offer insights into the pathogenesis of GC and reveal potential therapeutic targets. In addition, some genes are highly expressed in tumours but lead to a better prognosis. Understanding the cause of this abnormal phenomenon may provide new insights into the molecular mechanisms underlying cancer progression. Discovering specific genes related to GC in the PI3K-AKT pathway and identifying their interacting factors could increase the identification of GC risk factors. This will facilitate the molecular analysis of GC, thereby providing insights into its clinical treatment and prognosis.

In recent years, progress in the development of sequencing technologies and bioinformatic tools has shed light on understanding GC^10,11^. Many specific molecular markers were identified by analysing the sequencing data, providing a novel understanding of the mechanism and diagnosis of GC and introducing new treatments for GC. However, tumour heterogeneity poses a huge obstacle in regard to improving the diagnostic accuracy and precision of prognostic prediction. Therefore, it is necessary to identify effective molecular markers and targets at the single-cell level. High-throughput single-cell RNA sequencing (scRNA-seq) can reveals tumor heterogeneity, early biomarkers, rare clones, TME reprogramming, and metastatic mechanisms, guiding precision therapy, and provide an effective method that enables high-throughput profiling of gene expression at the single-cell level and dissect the complex cellular ecosystem of gastric tumors, enabling the identification of: (i) transcriptional programs in individual malignant cells, (ii) stromal cell subpopulations, and (iii) cell-cell communication networks that bulk sequencing cannot resolve^12^. To elucidate the transcriptome characteristics and interactions between cancer cells and microenvironmental components, scRNA-seq has been successfully used to decipher the ecosystem of GC, dissect and discover potential tumour target biomarkers of interest, and enrich relevant data on the single-cell transcriptomes of GC.

In this study, we identified the hub gene, *Ephrin A4* (*EFNA4*), in the PI3K-AKT pathway associated with GC using transcriptome data from the Gene Expression Omnibus (GEO) and The Cancer Genome Atlas Project (TCGA) databases. *ETS Proto-Oncogene 1* (*ETS1*) was identified as a key regulatory antagonist of EFNA4. Functional validation demonstrated that ETS1 actively suppresses EFNA4-mediated tumor suppression in gastric cancer, establishing a novel inhibitory axis in GC pathogenesis. The clinical relevance of the tumour cell-based signature was also determined, and the immune landscape underlying the signature was further analysed. We obtained GC single-cell RNA sequencing (scRNA-seq) data from accessible databases and then distinguished between different cells in the tumour tissue and sub-clusters of tumour cells. The expression patterns of the hub genes in the cells were identified at the single-cell level. Based on the hub genes and marker genes of their specific subcellular clusters, we constructed a tumour-based risk signature for GC. The prognostic value of the prognosis prediction model was verified using TCGA database to facilitate the clinical application of tumour cell features in the prognosis of GC. This study provides new insights into the pathophysiology of GC, thus leading to tailored treatments and improved outcomes for patients with GC. Despite EFNA4 showing oncogenic effects in liver and lung cancer^13,14^, its dual role in GC (tumor suppressive and improved prognosis) remains unclear and requires further exploration.

## Materials and methods

### Data collection

We collected a cohort of 373 GC and 32 normal gastric samples from The Cancer Genome Atlas (TCGA) database (https://tcga-data.nci.nih.gov/tcga/). Normalised RNA sequencing data and the clinicopathological details of the samples were obtained. Additionally, RNA sequencing datasets (GSE79973 and GSE19826) from the Gene Expression Omnibus (GEO) repository (https://www.ncbi.nlm.nih.gov/geo/) were downloaded. The GSE79973 dataset encompasses transcriptomic data from 10 cancerous and 10 normal tissues obtained from 10 patients with GC, whereas the GSE19826 dataset consists of transcriptomic data from 12 cancerous and 12 normal tissues obtained from 12 patients with GC^15,16^.

Moreover, we used the dataset GSE150290 from the GEO database for in-depth single-cell RNA sequencing analysis of GC, including samples from diffuse GC biopsies^17^. These samples were processed using 10× Genomics technology alongside the Illumina HiSeq 2500 sequencing platform, comprising 50,692 cells from biopsy samples from seven early gastric cancer (EGC) patients and 45,306 cells from biopsy samples from seven advanced gastric cancer (AGC) patients (Supplementary Table 1).

### Human bulk RNA sequencing analysis

Platform-specific normalisation methods were used (RNA-seq pipeline for TCGA with processing of raw data normalized by FPKM, and Limma’s normalizeBetweenArrays for the GEO microarray data). The `DESeq2` R package (version 1.38.3) and `limma` package in R^18^ were employed to analyse differentially expressed genes (DEGs) in the TCGA and GEO datasets respectively^19^. The criteria set for identifying DEGs in the GSE79973 dataset included an absolute logFoldChange (logFC) greater than 0.1 and a *p*-value below 0.05. In the GSE19826 dataset, DEGs were defined as those exhibiting an absolute logFC exceeding 0.1 with a *p*-value below 0.05. The analysis focused on comparing the two clusters that were grouped based on the median values of ETS1 and EFNA4 expression. The volcano plot shows the DEGs between the two clusters, with a logFC greater than 0.1 and a *p*-value below 0.05. DEGs were subsequently used for enrichment analysis.

### Bioinformatics prediction

Potential transcription factors (TFs) binding sites of EFNA4 were predicted using the JASPAR database^20^. Pearson’s correlation coefficient was used to quantify the relationship between EFNA4 and ETS1 expression. The correlation between EFNA4 and ETS1 was verified using TCGA-STAD datasets.

### Human protein atlas analysis

The Human Protein Atlas (HPA) database (https://www.proteinatlas.org/) was used to examine the expression of EFNA4 by immunohistochemical staining of GC. Tumour cells from the upper stomach gastric adenocarcinoma were obtained from a 69-year-old male patient, and normal tissue was obtained from a 72-year-old male. Additionally, we investigated the expression of ETS1 by immunohistochemical staining of gastric adenocarcinoma in the upper stomach of a 71-year-old male patient and normal upper stomach tissue of a 68-year-old male.

### Survival analysis

Univariate Cox model hazard ratio analysis was used to identify genes linked to overall survival (OS) in GC, and the forestplot package was used to visualise the hazard ratios. Differential gene expression analysis between the RNA-seq data of GC tissues and adjacent normal tissues was conducted to identify survival-related genes. Expression levels of EFNA4 and ETS1 were evaluated at different stages of GC. Survival curves for EFNA4 and ETS1 in GC were generated using the Kaplan–Meier method, and differences were assessed using the log-rank test^21^. To gauge the diagnostic accuracy of EFNA4 and ETS1 in GC, receiver operating characteristic (ROC) curve analyses were performed using the “ROCR” R package^22^. ROC curves demonstrated the effectiveness of a binary classification system across various decision thresholds, where death was considered a positive outcome and survival was considered a negative outcome.

### Cell culture and reagents

Human embryonic kidney 293 T (HEK293T) cells were acquired from the American Type Culture Collection (ATCC). Human gastric cancer cells (MGC803 and SGC7901) were obtained from the Cell Bank of the Type Culture Collection of the Chinese Academy of Sciences (Shanghai, China). All cells were cultured in Dulbecco’s modified Eagle’s medium (DMEM; Gibco, Grand Island, NY, USA) or Roswell Park Memorial Institute 1640 (RPMI-1640) medium supplemented with 10% foetal bovine serum (FBS; Gibco), 100 IU/mL penicillin, and 100 µg/mL streptomycin, maintained at 37 °C in a humidified incubator with a 5% CO_2_ atmosphere. The MycoAlert Mycoplasma Detection Kit (Lonza) was used to test the Mycoplasma contamination of cells.

### Plasmid construction and cell transfection

A 932-bp fragment of EFNA4 promoter region was amplified via PCR from genomic DNA using primers (EFNA4-promoter-FP: 5′-CCGGTACCTGAGCTCGCTAGCggtaccGGTTCCAGGTTTGGC-3′; EFNA4-promoter-RP: 5′-TCCGAAGACTCATTTAGATCTctcgagAAGTTGCGGAAAGGG-3′), and subsequently cloned into the PGL-4.20-TK luciferase reporter vector via homologous recombination using a ClonExpress II One Step Cloning Kit (Cat# C112; Vazyme, Nanjing, China). To construct ETS1 and EFNA4 protein expression plasmids, total RNA was isolated from HeLa cells using TRIzol reagent (Cat# 15596026; Invitrogen, Carlsbad, CA, USA), and the cDNA template was obtained via reverse transcription using a PrimerScript RT reagent kit (Cat# RR037B, Takara Bio Co., Ltd., Kusatsu City, Shiga Prefecture, Japan). ETS1 and EFNA4 CDS regions were amplified using specific primers. The forward primer (5′-GACGATGACAAGCTTatgagctactttgtggattctgct-3′) and reverse primer (5′-TCCTCTAGAGTCGACctcgtcggcatctggctt-3′) were for ETS1, and forward primer (5’-AAGGATGACGATGACAAGCTTaccaaaccggacctcgg-3’) and reverse primer (5’-TTCGGATCCTCTAGAGTCGACtcacagaattcgcagaagacga-3’) were for EFNA4, then the purified PCR products were inserted into the pCMV-3×Flag vectors via homologous recombination with ClonExpress II One Step Cloning Kit (Cat# C112; Vazyme, Nanjing, China). Either small interfering RNA (siRNA) or plasmids were transfected into proliferating cells using Lipofectamine 2000 (Invitrogen) following the manufacturer’s protocol.

### Dual-luciferase reporter assay

HEK293T cells were seeded in 24-well plates and cultivated until 70% confluence was reached 24 h before transfection. The cells were then co-transfected with the generated luciferase reporter constructs, empty PGL-4.20-TK vector, ETS1 overexpression plasmids, or pCMV-3× FLAG vectors, and the Renilla luciferase plasmid was defined as the relative luciferase activity. After 24 h, luciferase activity was measured using the Dual Luciferase Reporter Gene Assay Kit (cat#11402ES60; Yeasen, Shanghai, China) according to the manufacturer’s guidelines on the Dual-Luciferase Reporter Assay System. We calculated relative luciferase activity by normalizing Firefly luciferase activity to Renilla luciferase activity. For each experimental condition, we performed transfection in triplicate and conducted three independent reporter assays. Statistical analysis was performed using GraphPad Prism 8.0 (GraphPad Software, USA), with unpaired Student’s *t*-test for two-group comparisons (*n* = 2) and one-way ANOVA for multi-group comparisons (*n* ≥ 3).

### Functional enrichment and gene set enrichment analyses

DEGs were annotated using Gene Ontology (GO) and Kyoto Encyclopedia of Genes and Genomes (KEGG, www.kegg.jp/kegg/kegg1.html) analyses with the R package “Clusterprofiler”^23–25^. A *p*-value less than 0.05 was employed to establish the criteria for visualising enriched pathway results. In addition, Gene Set Enrichment Analysis (GSEA) was conducted using the same R package to investigate significant functional disparities of DEGs. Pathway enrichment was deemed significant based on the following criteria: an absolute normalised enrichment score (|NES|) greater than 1, a *p*-value less than 0.05, and a false discovery rate (FDR) *q*-value < 0.05.

### SiRNA transfection

To downregulate *EFNA4* expression in the GC cell lines, siRNA targeting *EFNA4* was synthesised at RiboBio Co., Ltd. (Guangzhou, China). The sequences were as follows: siEFNA4 (sense), 5′ -CAGGUGUCUGUCUGCUGCATT-3′, and non-targeting siRNA NC (cat#siN0000001-1–5, Guangzhou RiboBio, CO). Before transfection, SGC7901 and MGC803 cells were seeded onto cell plates and cultured in RPMI-1640 medium supplemented with 10% FBS for 24 h. After 48 h of transfection, the cells were collected for wound healing, transwell migration, and EdU assays.

### Wound-healing assays

As previously described^26^, SGC7901 and MGC803 cells were grown in 6-well plates as previously described. When the cells reached 85% confluence, they were transfected with 1 µg per hole of plasmids or 100 pmol per hole of siRNA and 2 µL of Lipofectamine 2000 for 6 h in transfection medium. After 48 h, the wound scrape of cells was created using a 100-µL sterile pipette tip, and the supernatant cells were washed away with phosphate buffer solution (PBS). Cells continued to be cultured in RPMI-1640 supplemented with 2% FBS, 100 µg/mL streptomycin, and 100 U/mL penicillin (Gibco BRL) at 37 °C under a humidified 5% CO_2_ atmosphere. The scratched areas were imaged at 0 and 12 h. The distances between the gaps were then calculated.

### Transwell migration assay

MGC803 and SGC7901 cells were grown in six-well plates and transfected with siRNA using Lipofectamine 2000 (Invitrogen). After 48 h, the cells were digested with trypsin (Gibco) and seeded in a 24-well plate in transwell chambers to detect cell migration and invasion. Cells were resuspended in RPMI-1640 without FBS and seeded into the upper inserts (1 × 10^5^ cells/well). After 6 h of cultivation, migrated and invaded cells were stained for 30 min with crystal violet buffer (10% ethanol and 0.2% crystal violet). The images were captured and quantified using a TCS SP8 confocal microscope (Leica).

### Cell proliferation assays and Immunofluorescence (IF) staining

SGC7901 and MGC803 cells were seeded in 96-well plates (1 × 10^3^ cells/well) and transfected with plasmids or siRNA for 48 h. Then, the cells were incubated with 10 µM 5-ethynyl-2′-deoxyuridine (EdU) for 4 h followed by being fixed for 30 min with 4% polyfluoroalkoxy (PFA) after cleaning with PBS. The Click-iT EdU kit (Thermo Fisher Scientific) was used to measure EdU levels in the cells. Nuclei were counterstained with DAPI-containing Fluoroshield (Sigma-Aldrich, Santa Clara, CA, USA) for 15 min. Fluorescent images were captured using a TCS SP8 confocal microscope (Leica). The images were analysed using ImageJ software 1.5.3^27^.

### Immune cell infiltration Estimation

To validate immune cell infiltration of GC, we incorporated three distinct analytical methodologies into the RNA expression data from 373 TCGA-STAD samples. We classified the GC samples based on EFNA4 and ETS1 expression profiles utilising the “ConsensusClusterPlus” R package^28^. We then quantified the immune and stromal cell fractions in the tumour microenvironment, resulting in ImmuneScore, StromalScore, and ESTIMATEScore^29^ using the ESTIMATE algorithm. These scores are directly related to the proportion of immune and stromal elements and inversely related to tumour purity, with higher values indicating increased component ratios in the tumour microenvironment.

The “CIBERSORT” R package transforms tumour tissue transcriptomic data to quantify the absolute abundance of 22 tumour-infiltrating immune cell types (TIICs), leveraging standard annotation files to outline gene expression features^30^. This approach delineates the proportional representation of diverse immune cells within the tumour microenvironment of GC. ssGSEA methodology was applied to gauge enrichment of the gene set^31^ by the “GSVA” R package. A comprehensive investigation of tumour-immune system interactions was conducted using TISIDB (http://cis.hku.hk/TISIDB/)^32^. We explored the correlation between tumour-infiltrating lymphocyte (TILs) abundance, immunomodulatory interactions, and EFNA4 and ETS1 expression. The interplay between gene expression and immune cell infiltration was revealed by calculating the Spearman correlation coefficients and their statistical significance, which were subsequently visualised as heatmaps.

### scRNA-seq data processing

As noted above, these two data sets, seven EGC and seven AGC, comprising 50k and 40k cells, respectively. Quality control and preprocessing of the scRNA-seq data were performed using the Seurat package in R (version 4.2.2) before batch-effect correction^33,34^. The preprocessing steps included: (1) Quality Control: Cells were filtered based on gene expression (standard deviation threshold: < 1), proportion of zero UMIs (threshold: > 90%), and the expression levels of mitochondrial and haemoglobin genes (threshold: >10% of total expression). Cells with UMI counts < 100 or > 20,000 were excluded. (2) We normalised gene expression data using the “LogNormalize” technique through Seurat’s “NormalizeData”. (3) Variable genes across cell populations were pinpointed with Seurat’s “FindVariableFeatures”. (4) Data were scaled and batch effects were addressed using the “ScaleData” function. (5) Principal component analysis initially reduced the data dimensions. To eliminate the batch effect of the scRNA-seq data, we conducted an integrated gene expression profiling analysis using Harmony software^35^. Then, uniform manifold approximation and projection (UMAP) was used to further refine these cells and cluster them based on a resolution parameter (RNA_snn_res.0.8) to identify distinct cell populations.

Marker genes identified in each cluster were compared to known cell marker genes from previous studies. The marker genes for the cell clusters include PTPRC (Immune cells), PLVAP, KDR, PTPRB (Endothelial Cells), CHGA, GAST, PROX1 (Enteroendocrine cells), MMP2, PDGFRA, MYL9, FN1, CAV1 (Fibroblasts), PGC (Chief Cells), MUC2, ITLN1, HES6 (Goblet Cells), MUC5AC, GKN1, GKN2, TFF1 (Pit Mucous Cells), EPCAM, CDH17, COL3A1, PDGFRB (Tumour Cells). The expression levels of genes in each cluster were depicted using dot plot visualisation.

### Pseudo trajectory analysis of single-cell sequencing data

Monocle2 (version 2.16.0)^36^ was used for expression analysis of EFNA4 and ETS1 during GC progression. We prepared a CellDataSet object incorporating preprocessed expression data with relevant metadata for in-depth analysis. The cells were ordered in pseudotime based on specific criteria (mean_expression ≥ 0.1 and dispersion_empirical ≥ 1 ^*^ dispersion_fit) for constructing the developmental trajectories. We reduced the dimensions and effectively mapped cellular development trajectories using the DDRTree method. Monocle2 identified and clustered genes that correlated with pseudotime (*q*-value < 0.01), thus highlighting the key genes involved in cellular transitions. Based on the pseudotemporal trajectory, cells were ordered and assigned to specific states.

### Analysis of intercellular communication in early and advanced gastric cancer

We analysed the intercellular communication among cell clusters in EGC and AGC using the CellChat package (version 1.6.1)^37^ to understand the ligand-receptor interactions of communication networks within the tumour microenvironment. The analysis was based on sea-normalised datasets, which were integrated into CellChat with the CellChatDB human database to map receptor-ligand interactions. The “computeCommunProb” function in CellChat calculated the probabilities of communication and provided a quantitative and qualitative analysis of cellular interactions based on both frequency and significance.

For depicting the signalling output of various cell clusters, we generated a heatmap using “netAnalysis_signalingRole_heatmap (cellchat, pattern = ‘outgoing’)”, highlighting the predominant senders within the communication network. To visualise cells primarily receiving signals, another heatmap was produced with “netAnalysis_signalingRole_heatmap (cellchat, pattern = ‘incoming’)”.

### LASSO regression for prognostic modelling

The construction of the prognostic model was based on the gene signatures within the tumour or endothelial cell clusters and the expression of EFNA4 and ETS1 using the FindAllmarker function of the Seurat package, with the parameters set to min. pct = 0.25 and logfc.threshold = 0.25. A univariate Cox regression model was used to detect DEGs in tumours or endothelial cells that were significantly correlated with patient survival outcomes. Following this, the LASSO regression analysis refined our selection of robust predictors of these outcomes^38^ through multivariate stepwise Cox regression. The risk score for each patient was computed using the formula: risk score = Σ (coefficient_mRNAn * expression level_mRNAn), enabling us to assess the relationship between risk scores and patient prognoses. Patients with stomach adenocarcinoma (STAD) were stratified into high- and low-risk groups based on the median risk score, and Kaplan–Meier survival curves were plotted to delineate differences in survival between these groups. The prognostic accuracy of our model was evaluated using time-dependent ROC curve analysis over 1, 3, and 5 years using the “Kaplan–Meier survival ROC” package, and the area under the ROC curve (AUC) was used to gauge the performance. The effectiveness of the models was visually presented through risk score distributions, patient survival status, and gene signature heatmaps. Multivariate Cox regression analyses were conducted to confirm that the risk score was an independent prognostic factor.

### Statistical analysis

All statistical analyses were conducted using the R software (version 4.2.2)^39,40^. Cox regression models, both univariate and multivariate, were applied using the “Survival” and “Tidyverse” packages in R. The *t*-test or Wilcoxon test was used for quantitative data, whereas Fisher’s exact or Pearson’s chi-squared tests were used for categorical data comparisons. Between-group differences were assessed by Student’s *t*-test (two groups) or one-way ANOVA (≥ 3 groups). Pearson’s correlation coefficients were computed, adhering to statistical correlation guidelines, to assess the co-expression correlation of the two proteins. The Kaplan–Meier method was employed to generate survival curves, which were then assessed using log-rank tests. Statistical significance thresholds were set at * *p*-value < 0.05, ** *p*-value < 0.01, *** *p*-value < 0.001, and **** *p*-value < 0.0001.

## Results

### Identification of EFNA4 as a key factor in GC pathogenesis

**Fig. 1:**
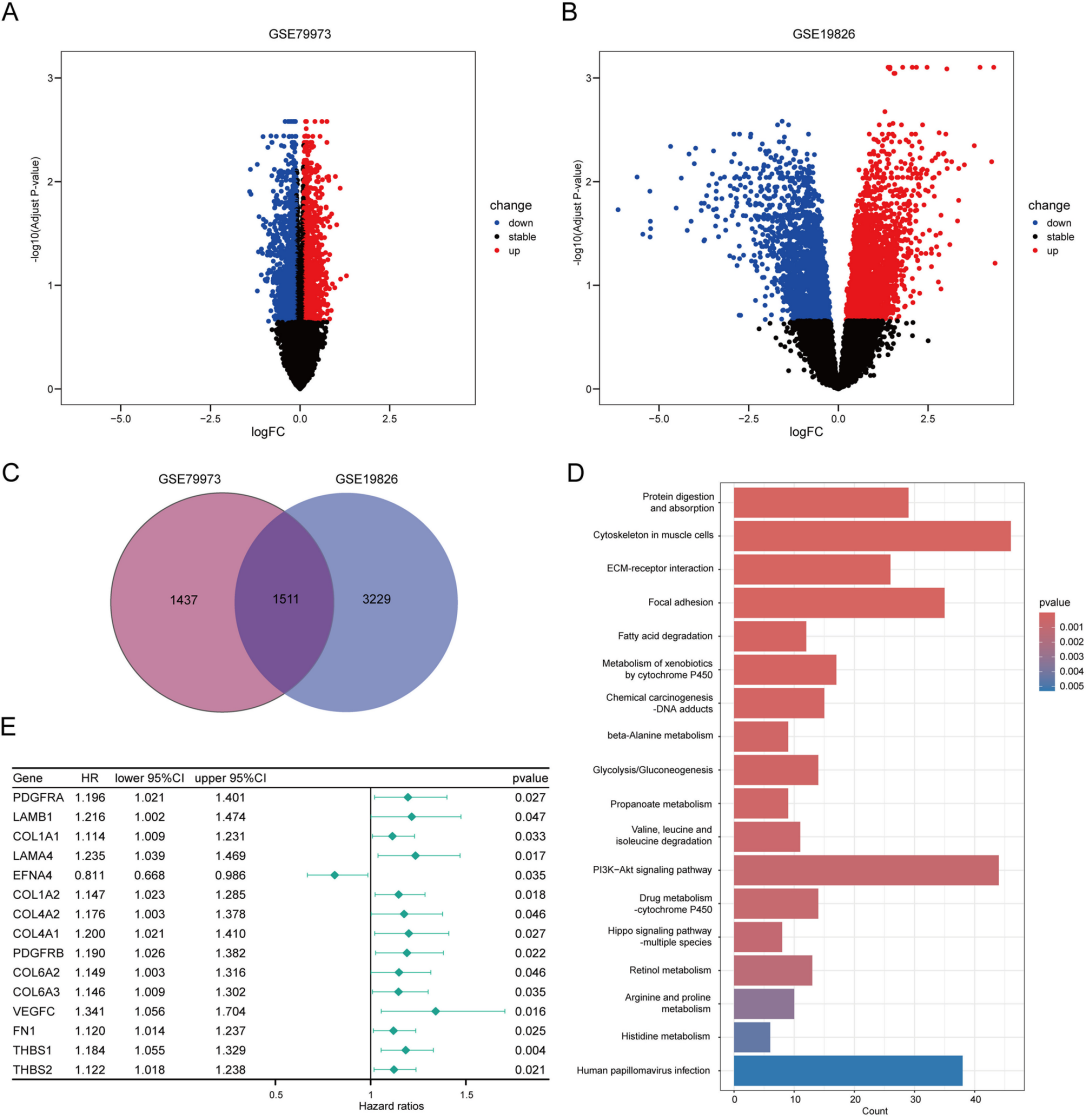
EFNA4 is established as a crucial modulator in the development of gastric cancer (GC) through analysis of differentially expressed genes (DEGs) and Kyoto Encyclopedia of Genes and Genomes (KEGG, www.kegg.jp/kegg/kegg1.html) pathway enrichment. Scanning differentially expressed GC-related DEGs in (**A**) GSE79973 and (**B**) GSE19826 datasets. logFoldChange (logFC) > 0.1, *p*-value < 0.05. (**C**) Venn diagram of overlapping DEGs from GSE19826 and GSE79973. (**D**) KEGG analysis of common DEGs of the two datasets. (**E**) Univariate Cox Regression for DEGs and patients’ survival. CI, confidence interval; HR, hazard ratio.

Two datasets (GSE79973 and GSE19826) were obtained from the GEO database (https://www.ncbi.nlm.nih.gov/geo/). To identify genes with significant expression changes in gastric tumour tissues compared to adjacent non-tumour tissues, we applied thresholds of logFC > 0.1 and *p*-value < 0.05, identifying 2,948 DEGs for GSE79973 (Fig. 1A, Supplementary Table 2), and thresholds of logFC > 0.1 and *p*-value < 0.05, identifying 4,740 DEGs for GSE19826 (Fig. 1B, Supplementary Table 3).

To refine the key factors in GC pathogenesis, we constructed a Venn diagram to determine the common DEGs between these two datasets, and 1,511 genes were obtained (Fig. 1C, Supplementary Table 4). Subsequently, we conducted a KEGG pathway analysis of the common genes (Fig. 1D, Supplementary Table 5). The most enriched pathways were associated with the occurrence and development of cancer, such as the ECM-receptor interaction, Hippo signalling pathway, retinol metabolism, and PI3K-AKT signalling pathway. Genes in the PI3K-AKT pathway are frequently dysregulated in human cancers, including GC, and affect the autophagy, EMT, apoptosis, chemoresistance, and metastasis of cancer cells. We found that 44 genes were enriched in the PI3K-AKT signalling pathway. We employed univariate Cox regression to determine the association between clinical factors and these 44 genes, 15 of which were associated with OS. Notably, only EFNA4 exhibited a hazard ratio (HR) < 1 (Fig. 1E). According to the RNA-seq data from TCGA-STAD, the mRNA expression levels of COL6A3, COL1A1, COL1A2, PDGFRB, THBS2, COL4A1, COL4A2, VEGFC, FN1, LAMB1, and EFNA4 were significantly higher in cancer tissues compared to normal tissues (Figure S1). These findings emphasise the potentially unusual involvement of EFNA4 and its regulatory network in the pathogenesis and progression of GC.

### EFNA4 overexpression in GC was correlated with better prognosis

**Fig. 2:**
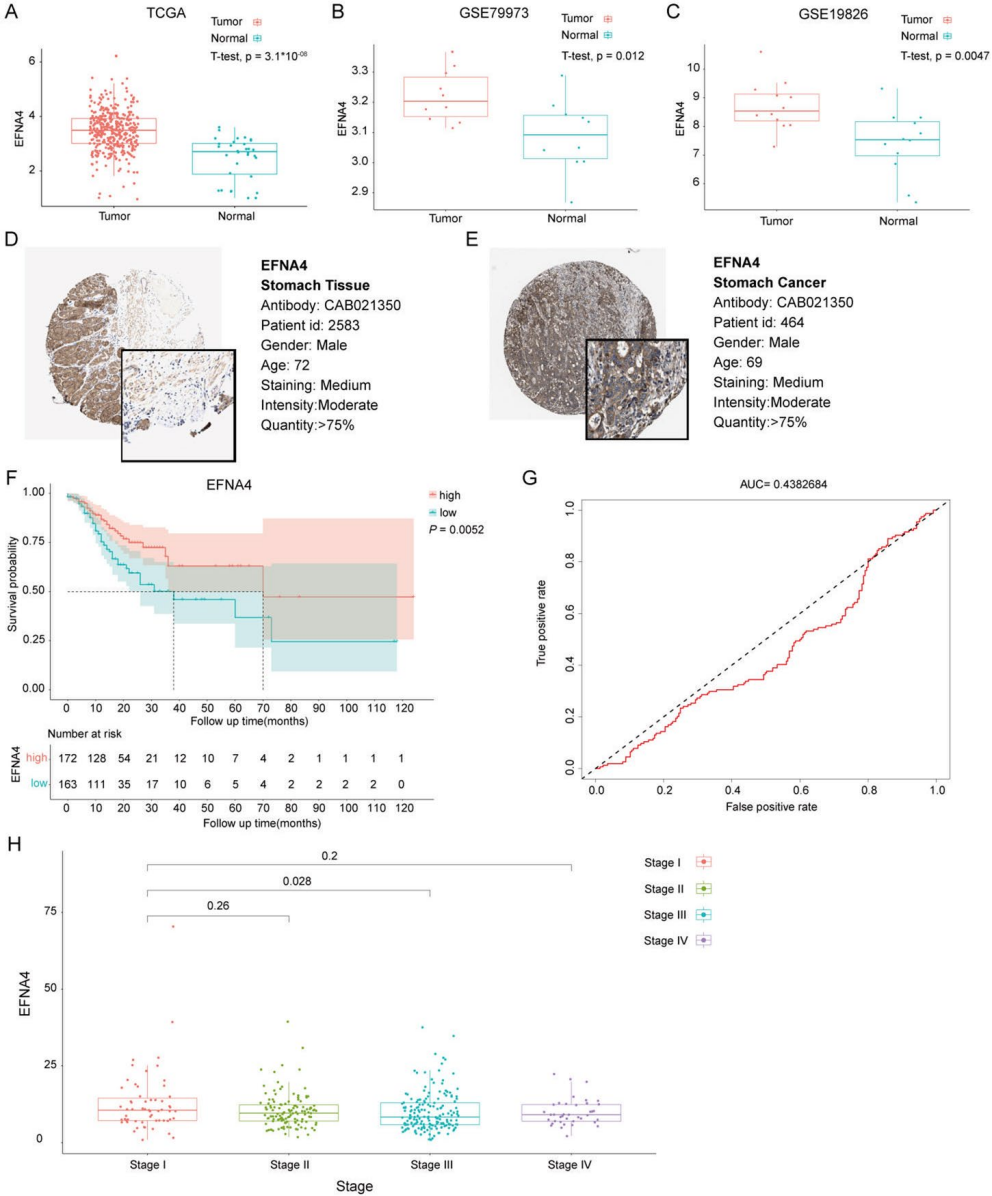
Examination of EFNA4 expression dynamics and intracellular distribution in GC. The expression of EFNA4 between gastric cancer tissues and normal tissues at the mRNA level in (**A**) The Cancer Genome Atlas (TCGA), (**B**) the GSE79973, and (**C**) the GSE19826 datasets. Immunohistochemical (IHC) of EFNA4 with the EFNA4-specific antibody CAB021350 between (**D**) normal and (**E**) gastric cancer specimens in the Human Protein Atlas (HPA) dataset. (**F**) The overall survival (OS) curves of EFNA4 in patients with GC. (**G**) The predictive performance of EFNA4 as a diagnostic marker using the ROC curve in the TCGA dataset. (**H**) The correlation analysis of EFNA4 expression and clinical stages of GC. Unpaired Student *t*-test was used for statistical analysis.

Using a one-sample, two-tailed *t*-test, we analysed RNA-seq data from the TCGA-STAD, GSE79973, and GSE19826 databases to compare the expression levels of EFNA4 between tumour and normal tissue samples. The analysis revealed a significant overexpression of EFNA4 in GC tissues (Fig. 2A-C). Using the HPA database for immunohistochemical assessments, EFNA4 was found to predominantly stain the cytoplasmic/membranous region of glandular cells and was upregulated in gastric adenocarcinoma tumour tissues compared to that in normal stomach tissues (Fig. 2D and E). Furthermore, we conducted Kaplan–Meier survival and ROC curve assessment analyses to evaluate the prognostic potential of EFNA4 in GC. Survival analysis indicated that patients with low EFNA4 expression had a poorer OS (Fig. 2F). We then performed ROC curve analyses to assess whether EFNA4 expression aided GC detection; the area under the curve (AUC) was 0.438, less than 0.5 (Fig. 2G). In addition, as the stages progressed, the expression level of EFNA4 in stage III was significantly lower than that at stage I (Fig. 2H). To further detect whether EFNA4 did influence cancer cell proliferation and motility abilities, wound healing, EdU, and Transwell assays were performed. EFNA4 siRNA treatment significantly promoted the migration and proliferative ability of both SGC7901 and MGC803 cells (*p*-value < 0.01) (Figure S2), as expected. Overall, these results suggest that patients with higher EFNA4 expression levels have an excellent prognosis.

### ETS1 was a potential regulatory factor for EFNA4 expression in GC

**Fig. 3:**
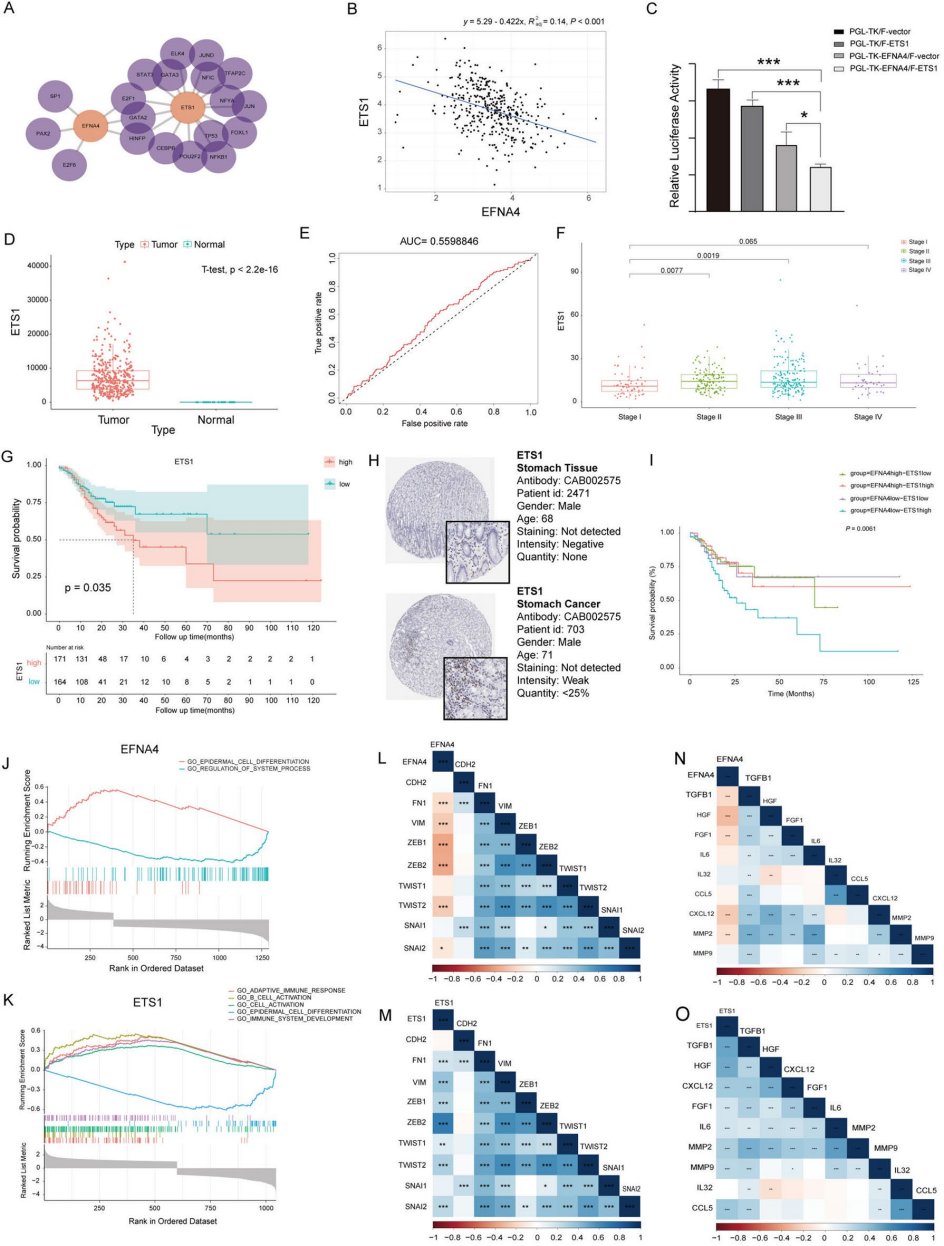
ETS1 was negatively correlated with EFNA4 in gastric cancer. (**A**) The potential transcription factors that bind to the EFNA4 gene based on the data from the JASPAR database. (**B**) The expression pattern of EFNA4 and ETS1 in the TCGA-STAD dataset. (**C**) Verification of ETS1-induced inhibition of EFNA4 transcription via dual luciferase reporter assay. The assay quantifies the inhibitory effect of ETS1 on the luciferase activity governed by the EFNA4 promoter in HEK293T cells. HEK293T cells were co-transfected with luciferase reporter plasmids driven by a human (pGL4.20-pEFNA4) promoter and ETS1 expression plasmids or control vectors. The ratio of firefly luciferase to Renilla activity was defined as the relative luciferase activity. The data represent fold increases (mean ± S.E. of triplicate experiments) relative to empty vector-transfected values. Student *t*-test was performed to determine the significance of the difference between each paired group. (**D**) ETS1 mRNA expression in gastric cancer and normal tissues. (**E**) Predictive performance of ETS1 as a diagnostic marker using the ROC curve in the TCGA dataset. (**F**) The correlation analysis of ETS1 expression and clinical stages of GC. Unpaired Student *t*-test was used for statistical analysis. (**G**) Kaplan–Meier analysis revealed the correlation of ETS1 expression and overall survival (OS) time. (**H**) IHC of ETS1 with the ETS1-specific antibody CAB002575 between normal specimens and gastric cancer tissues in the HPA dataset. (**I**) Survival analysis based on the expression patterns of EFNA4 and ETS1. GSEA-enriched pathways for dysregulated (**J**) EFNA4-related and (**K**) ETS1-related DEGs. The correlation of (**L**) EFNA4, or (**M**) ETS1, and mesenchymal stem cell and EMT markers. Heatmap of correlation coefficients between (**N**) EFNA4 or (**O**) ETS1 and various CAF secretion factors. (*p-value < 0.05, ** *p*-value < 0.01, *** *p*-value < 0.001).

To further identify the factors regulating the expression of EFNA4 in GC, we analysed these TFs using the JASPER website. We predicted the binding of TFs to EFNA4 and identified multiple potential binding TFs, including ETS proto-oncogene 1 (ETS1) (Fig. 3A). Spearman’s correlation analysis revealed a negative correlation between ETS1 and EFNA4 expression in a cohort of 407 GC samples from the TCGA Cancer Atlas (Fig. 3B). A dual-luciferase reporter assay was used to verify that ETS1 inhibited EFNA4 transcription (Fig. 3C). By applying a one-sample, two-tailed *t*-test to the RNA-seq data of STAD from the TCGA database, we observed a significantly higher expression of ETS1 in GC tissues than in normal tissues (Fig. 3D). The ROC curve was used to predict the survival of patients with STAD, and the AUC was 0.560 (Fig. 3E). ETS1 expression was significantly associated with cancer stage, and ETS1 expression in stage III was significantly amplified compared to that in stages I and II (Fig. 3F). Kaplan–Meier curves showed a significant difference in prognosis between patients with high-level ETS1 expression and those with low-level ETS1 expression in the cohort, with a more significant survival advantage for patients with low-level expression (Fig. 3G). ETS1 was located in the nucleus and was upregulated in gastric adenocarcinoma tumour tissues compared to normal stomach tissues (Fig. 3H). The patients were grouped according to the expression of EFNA4 and ETS1, and survival analysis was performed; patients with high expression of ETS1 and low expression of EFNA4 in the cohort showed the worst survival advantage (Fig. 3I).

To identify the signalling pathways distinguished by EFNA4 and ETS1 in GC, GSEA was performed to compare the high and low gene expression datasets according to the median value. The most significantly enriched signalling pathways are shown in Fig. 3J and K. Two and five significant GO pathways were significantly correlated with the expression of EFNA4 and ETS, respectively. The expression of EFNA4 significantly correlated with epidermal cell differentiation and the regulation of system processes. The expression of ETS1 significantly correlated with adaptive immune response, B cell activation, cell activation, epidermal cell differentiation, and immune system development. Notably, high expression levels of EFNA4 and ETS1 positively and negatively correlated with cell differentiation pathways, respectively. EFNA4 was inversely co-expressed with mesenchymal stem cell markers (FN1 and VIM) and transcription factors pivotal in EMT (ZEB1, ZEB2, TWIST2, and SNAI2) (Fig. 3L), and ETS1 showed a positive association with mesenchymal stem cell indicators (FN1 and VIM) and manifested affirmative correlations with EMT-related transcription factors (ZEB1, ZEB2, TWIST1, TWIST2, and SNAI2) (Fig. 3M). It hinted that EFNA4 and ETS1 had opposite effects on EMT, respectively. To further verify the role of EFNA4 and ETS1 in the process of EMT, TGF-β, HGF, FGF, SDF-1, IL-6, IL-32, CCL5, CXCL12, MMP-2, and MMP-9 were selected for correlation analysis with EFNA4 and ETS1, these genes are secreted by cancer-associated fibroblasts (CAFs) and then enable the EMT process. The result showed that EFNA4 and ETS1 were negatively and positively correlated with these genes, respectively (Fig. 3N and O). Moreover, ETS1 was positively co-expressed with vascular-(FLT1, KDR, CD34, and CDH5) and fibroblast-related genes (COL3A1, FBN1, and LUM), whereas EFNA4 exhibited the opposite pattern (Figure S3). Overall, these results indicate that ETS1 plays a crucial role in the progression of GC through the EMT pathway, and EFNA4 has the opposite effect in this process.

**Fig. 4:**
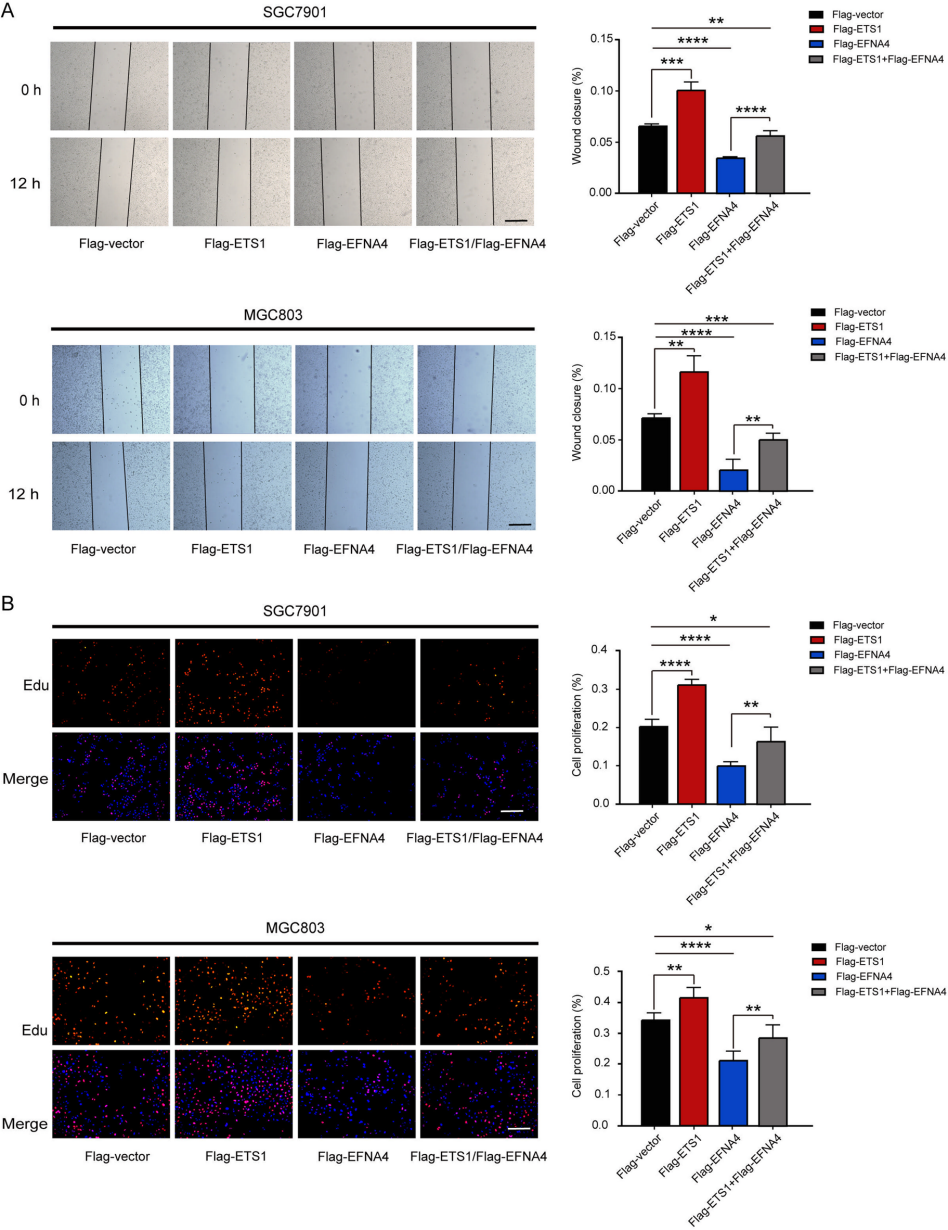
EFNA4 and ETS1 overexpression could affect the migration, invasive, and proliferative ability of gastric tumour cells in vitro. (**a**) Wound-Healing experiment in SGC7901 and MGC803 cells transfected with EFNA4 or/and ETS1. (**b**) EdU assays in SGC7901 and MGC803 cells after EFNA4 or/and ETS1 overexpression. Data are presented as the mean ± SEM from three independent experiments. Scale bar: 50 μm. Unpaired two-tailed *t*-test was used for statistical analysis between two groups. * *p*-value < 0.05, ** *p*-value < 0.01, and *** *p*-value < 0.001.

To detect the effects of ETS1 and EFNA4 on gastric cancer cells, we performed wound healing and Edu experiments. The results showed that the migration and proliferative ability of both SGC7901 and MGC803 cells were significantly inhibited by the overexpression of EFNA4 (*p*-value < 0.01), and promoted by the overexpression of ETS1. The ETS1 overexpression could promote the cell migration and proliferation ability inhibited by the up-regulation of EFNA4 (Fig. 4A and B). This further proves that EFNA4 and ETS1 play opposing roles in GC.

### The molecular function of the EFNA4 and ETS1 regulatory axis in GC

**Fig. 5:**
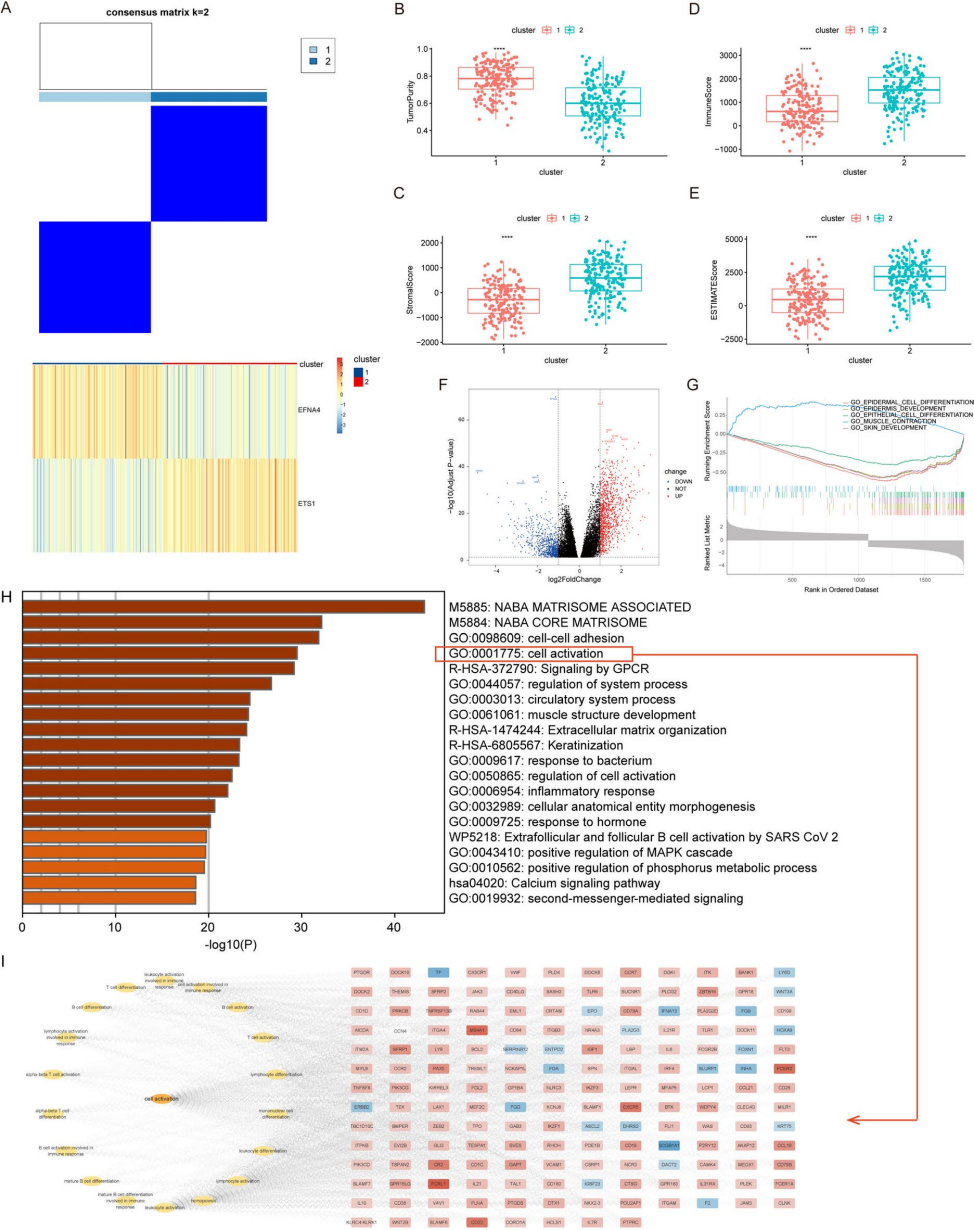
Analysis of differences between groups based on the expression of EFNA4 and ETS1. (**A**) Distinct clustering of 373 TCGA-STAD samples based on ETS1 and EFNA4 expression. The comparison of tumour purity (**B**), stromal score (**C**), immune score (**D**), ESTIMATE score (**E**) is conducted between the two clusters. (**F**) Volcano plot of differentially expressed genes (DEGs) between two clusters. (**G**) Gene set enrichment analysis for the DEGs. (**H**) The Bar graph shows pathway enrichment of DEGs via the Metascape database. Only the significant Gene Ontology terms (*p*-value < 0.05) are shown in rows. Terms with the prefix “hsa” from the KEGG database, the prefix “WP” from the WikiPathways, the prefix “R-HSA” from the Reactome, and “GO” are from the Gene Ontology Consortium. (**I**) The network diagram illustrates the sub-pathway and genes in the “cell activation” pathway.

To further understand the expression patterns of genes regulated by the EFNA4-ETS1 axis in GC, we performed consensus clustering on 373 TCGA-STAD samples based on the expression matrix of EFNA4 and ETS1, dividing the samples into two clusters. The optimal cluster number `K` for the TCGA-STAD dataset was determined to be 2 based on the maximum delta area and the rapid initial rise and subsequent plateau in the consensus CDF, suggesting a stable and biologically meaningful stratification of the data. The heatmap showed that Cluster 1 (*n* = 183) had low ETS1 and high EFNA4 expression, whereas Cluster 2 (*n* = 190) had high ETS1 and low EFNA4 expression (Fig. 5A and Supplementary Table 6).

Tumour purity, an intrinsic characteristic of GC, is associated with prognosis^41^. In Cluster 1, tumour purity was higher than that in Cluster 2 (*p*-value < 0.0001, Figure 5B), while the stromal, immune, and ESTIMATE scores were significantly lower (Fig. 5C-E). Cluster 1 was more active in the immune system than cluster 2.

We then analysed the differentially expressed genes between clusters 1 and 2 and identified 1109 upregulated and 768 downregulated genes (Fig. 5F, Supplementary Table 7). The functions of the DEGs were explored using GSEA to investigate the potentially modulated biological functions and signalling pathways. GSEA results revealed that upregulated DEGs were related to “MUSCLE CONTRACTION”, while downregulated DEGs were connected to “EPIDERMAL CELL DIFFERENTIATION”, “EPITHELIAL CELL DIFFERENTIATION”, “KERATINIZATION”, and “SKIN DEVELOPMENT” (Fig. 5G). Next, we used the Metascape database to explore the functional mechanisms underlying the DEGs. The top five biological effects were “NABA MATRISOME ASSOCIATED”, “NABA CORE MATRISOME”, “cell-cell adhesion”, “cell activation”, and “signaling by GPCR” (Fig. 5H). Of note, there are another 18 sub-pathways related to the immune cell activation, response, and differentiation, at the significantly enriched pathway “cell activation” (Fig. 5I). Notably, ETS1 has been reported to be associated with the immune microenvironment and immune cell function in multiple human tumours.

**Fig. 6:**
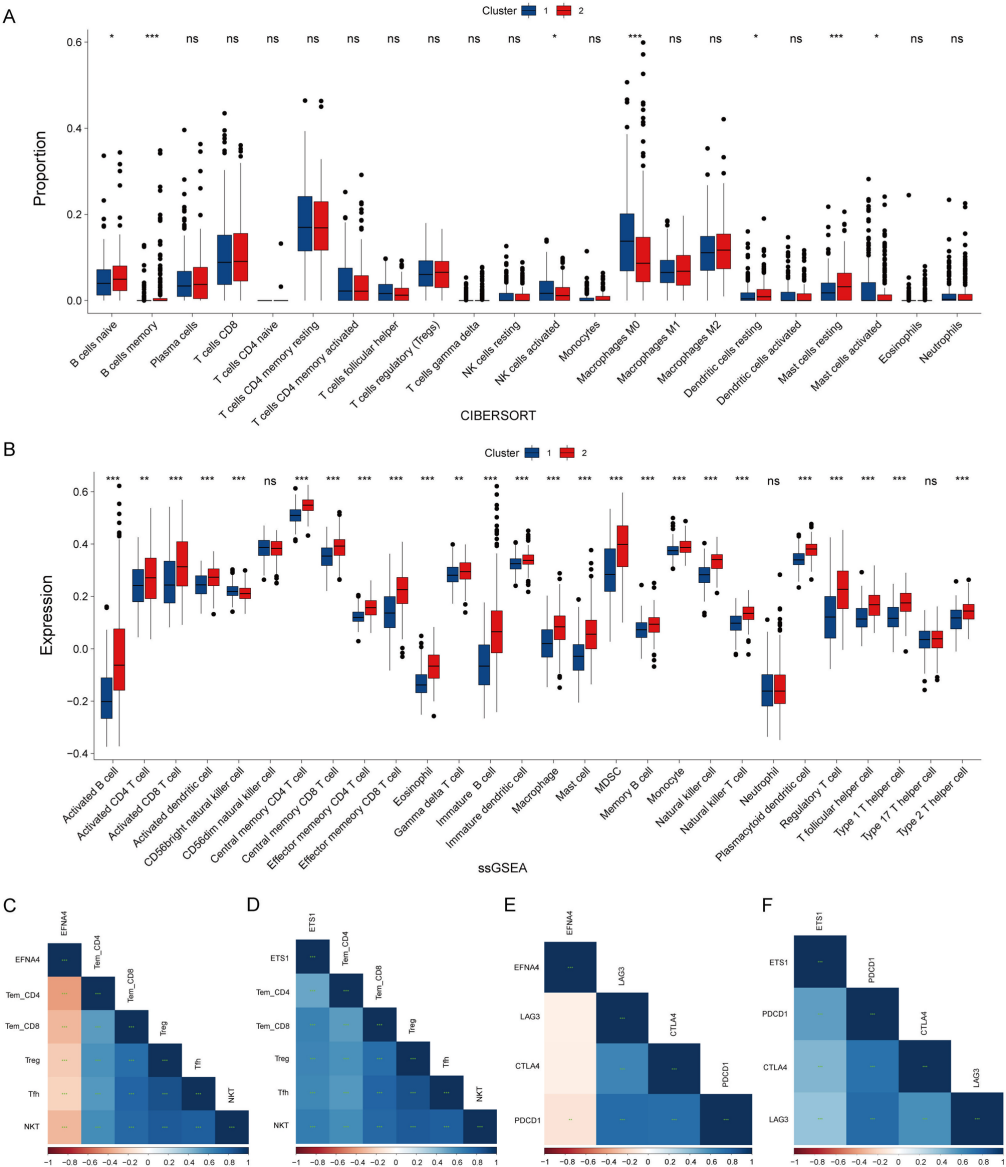
Correlation between gene expression and immune cell abundance. The comparison of immune cell proportion (**A**) and immune cell expression (**B**) is conducted between the two clusters (Groups with high and low expression of ETS1 and EFNA4). Heatmap illustrates the relationship between (**C**) EFNA4 or (**D**) ETS1 expression and various T cell subtypes. (**E**) Heatmap shows the correlation between (**E**) EFNA4 or (**F**) ETS1 expression and exhaustion markers LAG3, CTLA4, and PDCD1. Positive correlations are indicated by the blue shades, negative correlations by red shades, and the intensity of the colour reflects the strength of the correlation. Statistical significance levels are determined by the Wilcoxon test. (ns, not significant, * *p*-value < 0.05, ** *p*-value < 0.01, *** *p*-value < 0.001).

To explore the role of EFNA4 and ETS1 regulatory axis in the immune microenvironment of GC, we used the chi-square test to examine the available clinical data of patients with GC in Clusters 1 and 2. A statistically significant difference was observed between the two clusters in the T category of the TNM staging system and tumour stage (Supplementary Table 8). CIBERSORT analysis indicated that cluster 2 had higher proportions of naïve B cells, memory B cells, resting dendritic cells, and resting mast cells, whereas cluster 1 had higher proportions of activated NK cells, M0 macrophages, and activated mast cells (Fig. 6A). ssGSEA revealed that 24 immune cell subpopulations were highly expressed in cluster 2, whereas CD56 bright natural killer cells were highly expressed in cluster 1 (Fig. 6B).

**Fig. 7:**
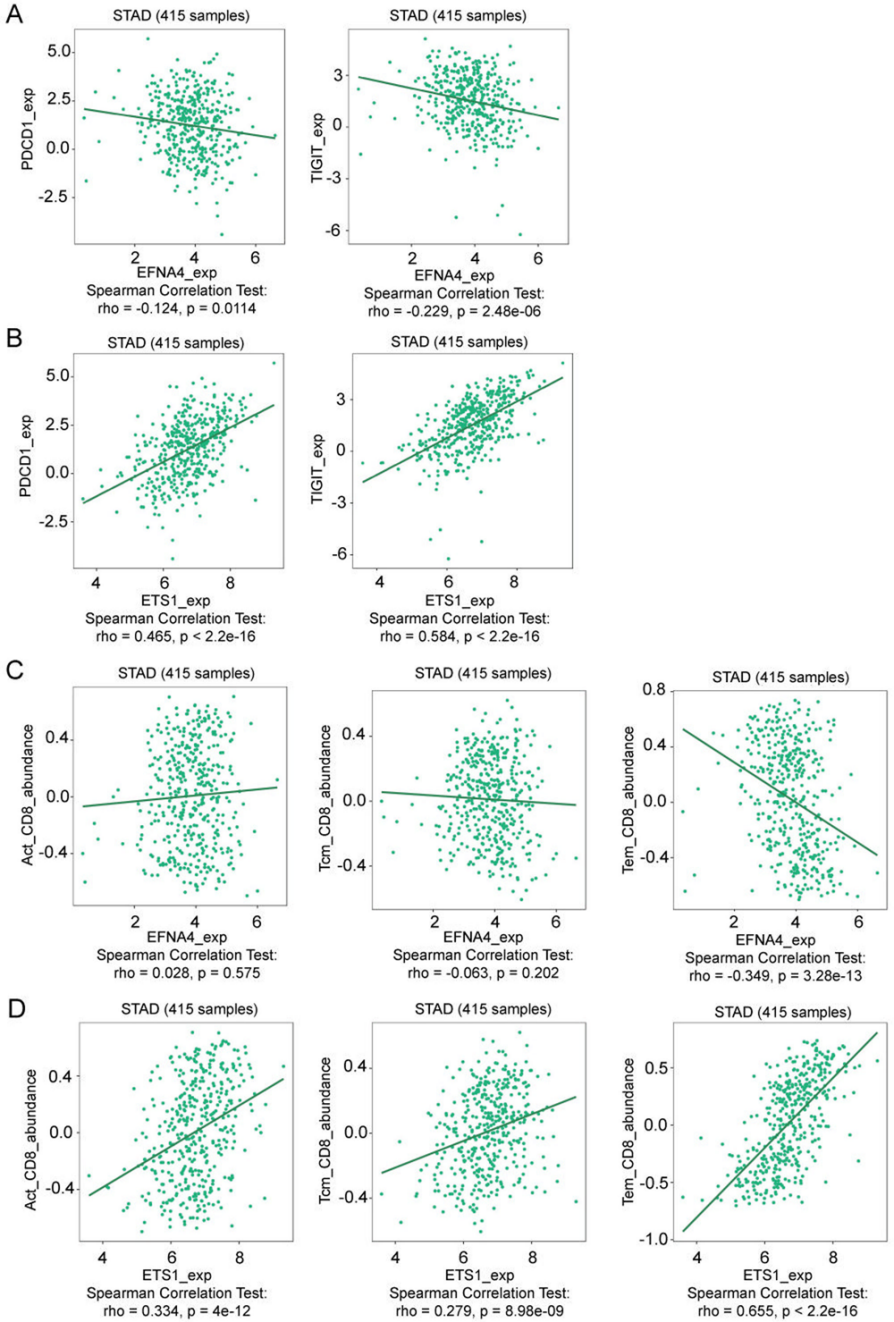
Roles of EFNA4 and ETS1 in the immune response of gastric cancer based on the TISIDB database. Correlation patterns of (**A**) EFNA4 and (**B**) ETS1 with various immune checkpoints. Relationships of (**C**) EFNA4 and (**D**) ETS1 with specific subsets of CD8 + T cells.

ETS1 positively correlated with immune cells, including Tem_CD4, Tem_CD8, Treg, Tfh, and NKT cells, whereas EFNA4 negatively correlated with these immune cells (Fig. 6C and D). ETS1 was positively correlated with the marker genes (PDCD1, CTLA4, and LAG3) of T cell exhaustion, and EFNA4 was negatively correlated with LAG3 (Fig. 6E and F). We then performed a thorough study using the TISIDB database to further elucidate immune checkpoint correlations and the relationship between EFNA4, ETS1, and tumour immunity in the context of GC. We evaluated the correlation between EFNA4, ETS1 and various immune checkpoints. Our results demonstrated a negative correlation between EFNA4 and the immune checkpoints, PDCD1 and TIGIT (Fig. 7A), whereas ETS1 was positively correlated with these checkpoints (Fig. 7B). According to the TISIDB database, EFNA4 was not correlated with activated CD8 + T cells and central memory CD8 + T cells but was negatively correlated with effector memory CD8 + T cells (Fig. 7C). ETS1 was positively correlated with activated CD8 + T cells, central memory CD8 + T cells, and effector memory CD8 + T cells (Fig. 7D). This evidence further substantiated the opposite effects of EFNA4 and ETS1 in the context of tumour immunity, playing an important role in immune cell-related pathways, and suggesting that they may affect the progression of GC through T cell exhaustion.

### The expression pattern of EFNA4 and ETS1 at the single-cell level

**Fig. 8:**
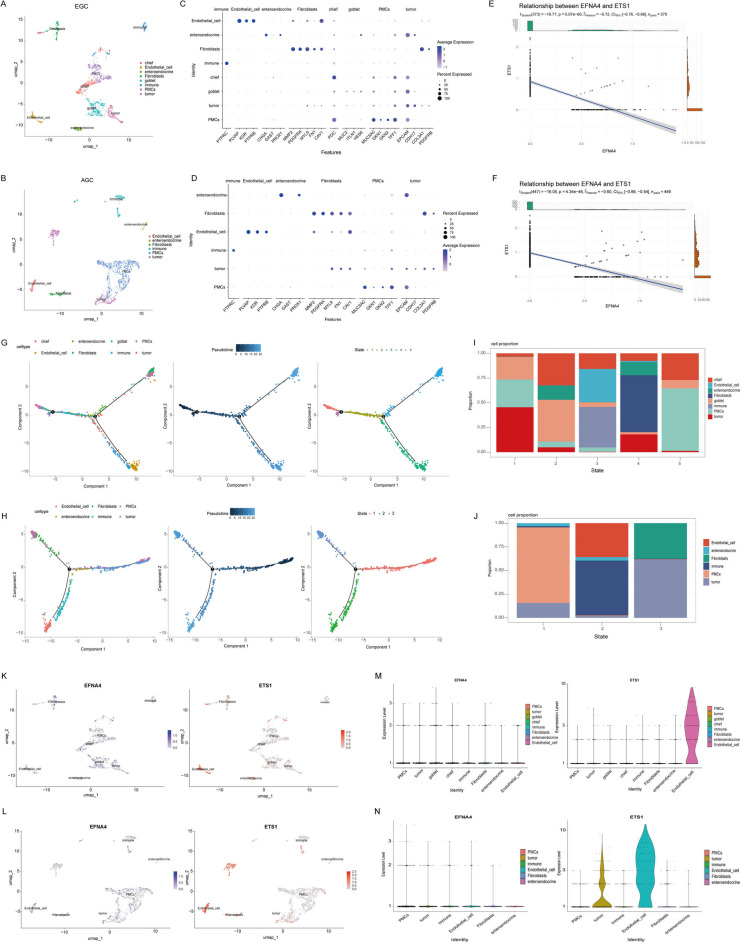
Expression pattern of EFNA4 and ETS1 in early gastric cancer and advanced gastric cancer at the single-cell level. UMAP visualization of 1,598 single cells from (**A**) early gastric cancer (EGC) tissues and 1,626 single cells from (**B**) advanced gastric cancer (AGC) tissues. The cell types were delineated by color based on the expression of specific marker genes, including chief cells, endothelial cells, enteroendocrine cells, fibroblasts, goblet cells, immune cells, pit mucous cells (PMCs), and tumour cells. The dot plot revealed the expression of marker genes of each cell type in (**C**) EGC and (**D**) AGC. Scatter plot with linear regression demonstrates the correlation between EFNA4 and ETS1 gene expression in (**E**) EGC and (**F**) AGC. Trajectory analysis of (**G**) EGC and (**H**) AGC cells, colour-coded by branch type (left), pseudotime (middle), and state (right). The bar chart represented the proportions of eight cell types across five states in (**I**) EGC and (**J**) AGC. UMAP scatter plots illustrated relative expression levels of EFNA4 and ETS1 in (**K**) EGC and (**L**) AGC. Violin plots displayed the distribution of EFNA4 and ETS1 expression in different cell types in (M) EGC and (**N**) AGC.

RNA-seq reflects changes at the tissue level, but cannot clearly distinguish between tumour and stromal cells in the tumour tissue. To further clarify the expression patterns of EFNA4 and ETS1 in GC tumours at the single-cell level, single-cell data (10x Genomics) of patients with early stage and advanced GC were downloaded from https://www.ncbi.nlm.nih.gov/geo/ (No: GSE150290). After processing the scRNA-seq data through cell cycle correction, normalization, dimensionality reduction, and clustering, we visualized the resulting cell populations using UMAP, which revealed distinct cluster distributions (Figure S4, Supplementary Tables 9 and 10). Based on cell-specific markers, all of the cells from the EGC and AGC tumour tissues were classified into eight and six categories, respectively. As illustrated in the uniform manifold approximation and projection (UMAP) plots, distinct marker genes typified EGC and AGC clusters. Chiefly, endothelial cells, enteroendocrine cells, fibroblasts, goblet, immune cells, PMCs, and tumour cells were identified in EGC, whereas endothelial cells, enteroendocrine cells, fibroblasts, immune cells, PMCs, and tumour cells were identified in AGC (Fig. 8A-D, Supplementary Tables 11 and 12). The expression of EFNA4 and ETS1 in EGC and AGC was negatively correlated (Fig. 8E and F). We then performed a pseudo-time trajectory analysis of the cells in the EGC and AGC to estimate the lineage relationships between the cell clusters. EGC cells are divided into five states, with tumour cells, goblet cells, and PMCs being the main cells in the initial state. The terminated state, state 5, consists of PMCs, chief cells, and goblet cells. The arrangement of AGC cells on the pseudo-timeline was divided into three states with the main components: (1) PMC and tumour cells, (2) endothelial and immune cells, and (3) tumour and fibroblast cells. Both EGC and AGC contained subclusters of tumour cells in different states (Fig. 8G-J). Subsequently, we analysed the expression levels of EFNA4 and ETS1 in EGC and AGC. The expression of EFNA4 was uniform in each cell cluster in EGC, whereas ETS1 expression was higher in endothelial cells. In AGC, EFNA4 was found to be slightly downregulated in all cells except PMCs, and was significantly downregulated in enteroendocrine cells and fibroblasts. The expression of EFNA4 and ETS1 is depicted in the UMAP and violin plots, and ETS1 was significantly upregulated in tumour and endothelial cells (Fig. 8K-N).

### The expression patterns of EFNA4 and ETS1 were different in the subclusters of AGC and EGC tumour cells

**Fig. 9:**
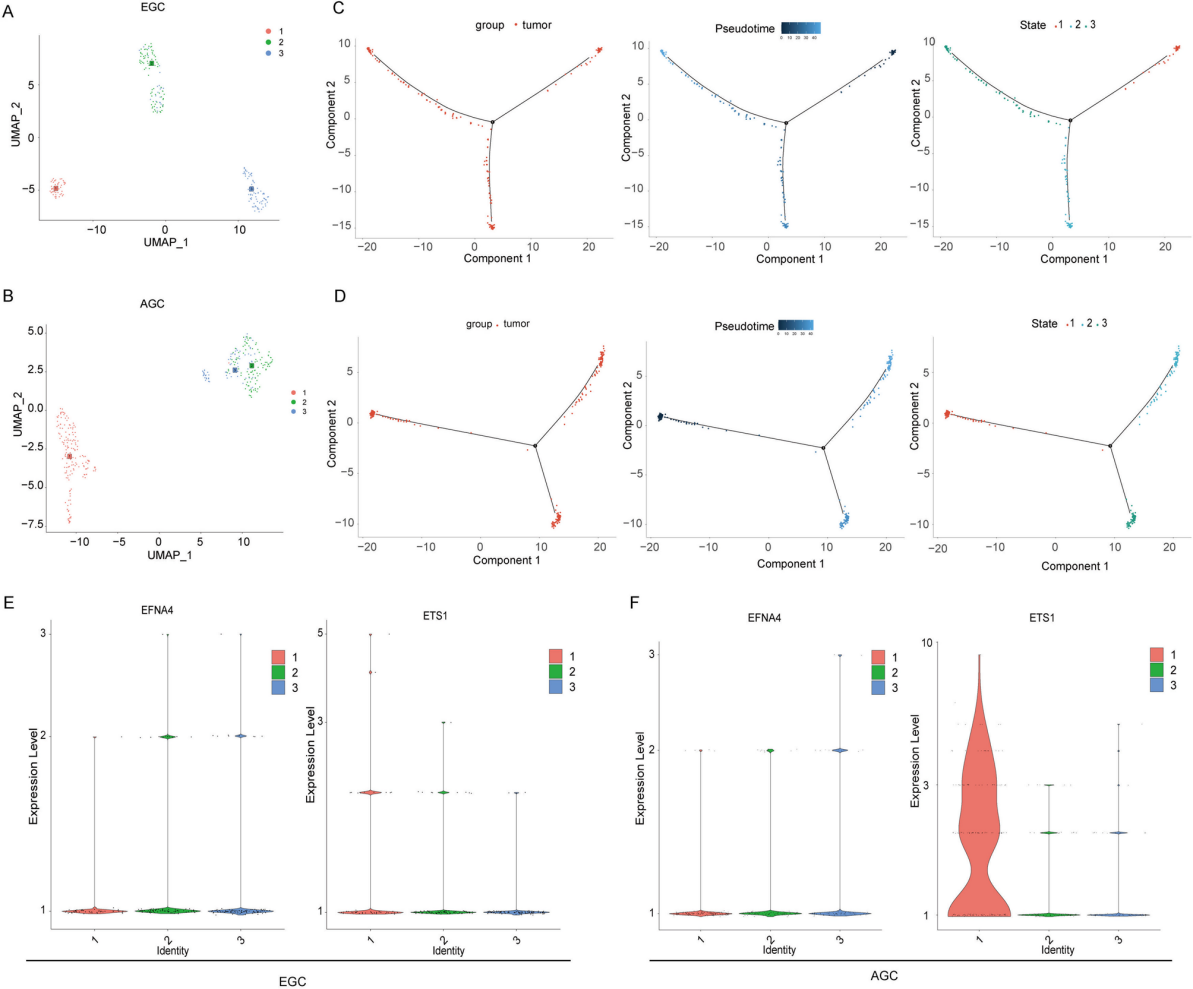
Unravelling the expression of EFNA4 and ETS1 in the subclusters of tumour cells of EGC and AGC. UMAP visualization of subclusters of tumour cells in (**A**) EGC and (**B**) AGC. (**B**) Trajectory analysis revealed the dynamic progression of tumour cells in (**C**) EGC and (**D**) AGC. Violin plots characterized the expression patterns of the EFNA4 and ETS1 in various states of (**E**) EGC and (**F**) AGC tumour cells.

The pseudotime analysis displays a valuable tool to explore tumour heterogeneity and to detect different intratumoural cellular states based on gene expression patterns. In order to further identify the expression of EFNA4 and ETS1 during tumour cell differentiation, we analysed EGC and AGC tumour cells. First, tumour cells were extracted from an scRNA-seq dataset of GC tissues isolated from seven patients with EGC and seven patients with AGC and weighed according to the expression levels of allele-specific Interaction Genes (ASIGs). The results showed that both EGC and AGC had three cellular subpopulations of tumour cells (Fig. 9A-D), suggesting a certain degree of tumour heterogeneity in EGC and AGC tumour cells. In tumour cells extracted from EGC and AGC scRNA-seq data, the expression of EFNA4 and ETS1 changed during pseudotime development. In EGC, the expression level of ETS1 in tumour cells in state 1 was high and decreased over time. The expression level of EFNA4 showed an opposite trend to that of ETS1. The expression patterns of ETS1 and EFNA4 in the AGC tumour cell subclusters were similar to those in the EGC tumour cell subclusters. Most notably, the percentage of tumour cells with high ETS1 and low EFNA4 expression was higher in AGC than in EGC (Fig. 9E and F).

Taken together, we have demonstrated that the expression of EFNA4 and ETS1 may influence the differentiation and characteristics of tumour cells, thereby affecting the progression of GC.

### Construction of prognosis prediction model and verify model validity

Given the changes in tumour cell clusters during GC progression and the CellChat results, the number and weight of outgoing signals of communication between tumour cells and immune cells were prominently increased (Figure S5). We chose the tumour cell cluster-related genes based on the single-cell data of EGC and AGC to construct a prognostic risk model.

**Fig. 10:**
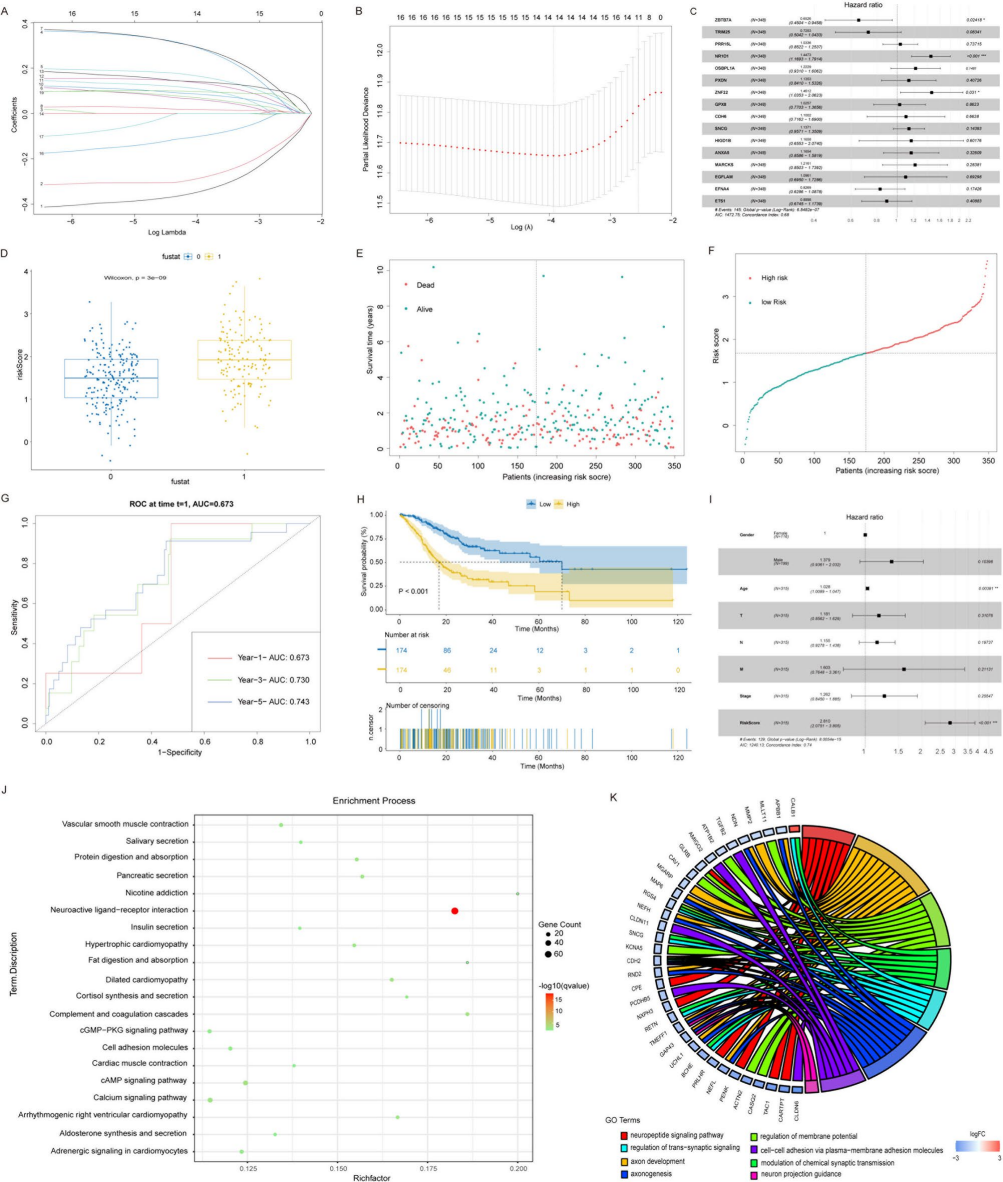
The development of a predictive model for tumour cell behaviour and the survival analysis associated with tumour progression. (**A**) The lasso coefficient profiles for key genes (EFNA4, ETS1, and tumour cell marker), with lines representing individual variable coefficients against log-transformed lambda values, where lambda is the lasso penalty parameter. (**B**) The cross-validation curve for optimal model selection in Lasso regression, using binomial deviance on the y-axis against log-transformed lambda values, where the dotted lines indicate the lambda value that minimizes error. (**C**) Forest plots showing the results of the univariate Cox regression analysis between the key genes and overall survival. (**D**)The box plot revealed that the survival outcomes were significantly different between high-risk and low-risk groups. (**E**) and (**F**) The scatter plot displaying the risk score, patients’ survival, and status for GC. The black dotted line divides patients into the low-risk group and the high-risk group. (**G**) Receiver Operating Characteristic (ROC) curves for a predictive model’s performance of risk score at 1, 3, and 5 years in the TCGA dataset. The Area Under the Curve (AUC) values quantify predictive accuracy at these intervals. (**H**) The Kaplan-Meier survival analysis was conducted between two groups stratified by the median risk score of low and high-risk groups in the TCGA dataset. (**I**) The multivariate Cox regression analysis evaluates the clinical characteristics and risk scores on survival. (**J**) The KEGG pathway enrichment analysis of differentially expressed genes (DEGs) between high- and low-risk groups. The dot size indicates gene count, and colour intensity denotes *p*-value significance. (**K**) The circular plot (chord diagram) of Gene Ontology (GO) enrichment analysis highlights the top eight GO terms of the DEGs.

Univariate Cox regression analysis was conducted to analyse 1,374 DEGs in EGC tumour cells and 1,204 DEGs in AGC tumour cells. By applying a screening criterion of *p*-value < 0.01, 6 tumour cell DEGs in EGC and 27 tumour cell DEGs in AGC were identified as associated with GC prognosis in the TCGA cohort, excluding any overlapping genes between EGC and AGC (Supplementary Tables 13 and 14). Subsequently, the LASSO algorithm was employed for specific OS gene range shrinkage (Fig. 10A and B). Sixteen genes, including EFNA4 and ETS1, and their regression coefficients were identified using Multivariate Cox Regression and were selected to calculate the risk score (Fig. 10C). A risk score model was constructed to associate patients with high-risk scores and poor survival outcomes (Fig. 10D). All patients with GC were divided into low- and high-risk groups based on median risk scores (Fig. 10E and F). The survival time of patients with STAD was evaluated using the ROC curve, and the AUCs for the 1-, 3-, and 5-year OS rates were 0.673, 0.730, and 0.743, respectively (Fig. 10G). The OS of patients with STAD significantly decreased with an increased risk score in TCGA database (*p*-value < 0.001, Fig. 10H). Multivariate Cox regression analysis demonstrated that the risk score and age were independently correlated with OS in TCGA cohort (*p*-value < 0.01) (Fig. 10I). This risk model was better for evaluating the prognosis of patients with GC than the prognostic model based on endothelial cell marker genes (Figure S6 and Supplementary Tables 15 and 16).

Subsequently, we conducted an analysis in order to identify the DEGs between the low- and high-risk groups. By setting stringent criteria requiring an absolute logFC greater than 1 and an adjusted *p*-value less than 0.05, we were able to successfully identify 1,141 DEGs. These genes were subjected to KEGG pathway and GO term enrichment analyses to elucidate their biological processes. KEGG pathway enrichment analysis showed that the DEGs were enriched in tumour-related signalling pathways, including the calcium signalling pathway, cAMP signalling pathway, and cell adhesion molecules, et al. (Fig. 10J). GO terms showed that the DEGs were enriched in the pathway of cell-cell adhesion via plasma-membrane adhesion, modulation of chemical synaptic transmission, and regulation of trans-synaptic signaling, et al. suggesting that activation of these pathways increases patients’ mortality risk (Fig. 10K).

## Discussion

GC is one of the most common digestive system tumours worldwide. Although multiple novel therapies, including chemotherapy, targeted therapy, and immune therapy, have been developed, the prognosis of GC has not significantly improved in recent years, particularly in patients with advanced-stage disease. Thus, identifying early marker genes for GC and constructing an effective tumour prognosis model will be helpful for the prevention and treatment of GC. However, the complex underlying mechanisms of GC limit the exploration of novel treatments to improve the prognosis of patients with GC, especially in the presence of tumour heterogeneity. Tumour heterogeneity is an important topic in tumour biology. Different GC subtypes have demonstrated substantial heterogeneity in both tumour cells and the microenvironment. Owing to the rapid development of biological technologies, tumour heterogeneity among individuals has been well decoded, which has mainly contributed to the selection of individual and specific chemotherapies and immunotherapies. In recent years, with advancements in sequencing technologies, massive amounts of tumour sequencing data have emerged, enabling a better understanding of the molecular changes involved in the occurrence and development of tumours. In this study, we explored the risk factors associated with GC and constructed a prognostic model for GC based on gene expression patterns using transcriptome and scRNA-seq data. To screen for novel potential regulatory factors of GC from the transcriptome data, we chose to use a less stringent threshold of 0.1 for logFC in order to define differentially expressed genes, which produced more significant genes. Although this approach may decrease the specificity, considering that this was the first step in this study, we prioritised sensitivity over specificity in our analysis.

The phosphatidylinositol 3-kinase (PI3K)/AKT signalling pathway is an intracellular signalling pathway of great importance in a range of cellular processes, including proliferation, metabolism, metastasis, and angiogenesis. It is hyperactivated or altered in many cancer types, influencing the induction of metastasis and EMT, thus leading to tumour progression, metastasis, and treatment resistance. The PI3K/Akt pathway is abnormally expressed in patients with chronic atrophic gastritis (CAG) and GC^42^, especially in *Helicobacter pylori*-positive patients. Moreover, activation of the PI3K/Akt pathway is involved in multidrug resistance during GC treatment. Thus, studying the expression and correlation of the PI3K/Akt pathway in GC is of great significance for the development, prognosis, and clinical success of targeted therapeutic agents for GC. In this study, EFNA4 was identified as a highly expressed gene in the PI3K/Akt pathway, which affected the progression of GC, but was correlated with a better prognosis. EFNA4 is a member of the ephrin (EPH) family and it belongs to a large subfamily of receptor protein-tyrosine kinases. Ephrins are categorised as ephrin A (EFNA), glycosylphosphatidylinositol-anchored ephrin B (EFNB), or transmembrane proteins^43^. The ephrin family plays important roles in the development and occurrence of cancer in humans. In previous studies, EFNA4 was shown to promote cell proliferation and tumour metastasis in hepatocellular carcinoma^13^. High levels of EFNA4 promote lung tumour cell proliferation and migration, and EFNA4 plays an oncogenic role in promoting lung cancer lymph node metastasis^14^. However, in GC, the relationships between EFNA family genes (EFNA1, EFNA2, EFNA3, EFNA4, and EFNA5), the immune microenvironment, and the IC_50_ of common chemotherapeutic drugs for GC were investigated. CD8 + T cell and dendritic cell infiltration were negatively correlated with EFNA4 expression. High EFNA4 expression was significantly associated with higher OS^44^. Many tumour cell genes exhibit expression and functional characteristics, such as CXCL11, which is highly expressed in colon cancer and is correlated with anti-tumour immunity and improved prognosis^45^. In addition, in vitro experiments showed that knockdown EFNA4 can promote the migration, invasion, and proliferation of GC cells. However, the molecular mechanisms underlying the changes in the expression of EFNA4 in GC still remain unclear, and the function of EFNA4 in GC requires further analysis.

To further identify the function of EFNA4 in GC, we predicted ETS1 binding sites within the EFNA4 promoter region using the JASPAR website. Both EFNA4 and ETS1 were overexpressed in GC tissues, with a negative correlation seen between their expression levels in GC tumour samples, suggesting a regulatory relationship. This finding was supported by the results of the dual-luciferase reporter assay. EFNA4 overexpression inhibited cell proliferation promoted by ETS1 upregulation. This inverse relationship implies antagonistic roles for these genes in tumour progression and immune response regulation. ETS1, an ETS family transcription factor, is involved in cell proliferation, apoptosis, differentiation, and migration, and is frequently overexpressed in various cancers, contributing to tumour progression and poor prognosis^46^. In previous studies, increased ETS1 expression has been associated with higher grading, poorer differentiation, and increased invasiveness in cancer cells. ETS1 expression is also linked to higher microvessel density and correlates with angiogenic factors VEGF, bFGF, and Ang2^47^. Among the TN/BLBC cell lines, those with a mesenchymal phenotype, such as MDA-MB-231 cells, exhibit the highest ETS1 levels^48^. This, along with high ETS1 expression in stromal cells such as CAFs and MSCs, suggests a relationship between ETS1 and the mesenchymal cellular phenotype^49^. Our study has emphasized the role of ETS1 in angiogenesis, fibroblast activation, and EMT in GC, corroborating its importance in malignancies. Conversely, EFNA4 overexpression inhibited angiogenesis, fibroblast activation, and EMT, with a negative correlation with mesenchymal stem cell markers and EMT transcription factors, suggesting a suppressive function. Given that the PI3K-AKT pathway can target ETS1 to promote tumor progression in multiple cancers, and ETS1 has been demonstrated to drive epithelial-mesenchymal transition (EMT), this study hypothesizes that EFNA4 may inhibit the PI3K-AKT signaling axis, thereby counteracting ETS1-mediated EMT. This mechanism may provide a novel therapeutic target for enhancing chemotherapy sensitivity in GC^50,51^. The scRNA-seq analysis showed that the preserved expression of EFNA4 in gastric gland mucous cells may reflect its unique role in the gastric mucosa, where it produces mucus as a protective layer against gastric acid, suggesting that its function in mucosal protection is maintained. Enteroendocrine cells, which secrete hormones that affect gastric motility, secretion, and absorption, may influence tumour growth and spread. EFNA4 downregulation in these cells may indicate the disruption of normal cellular signalling pathways. Cancer-associated fibroblasts (CAFs) in the tumour microenvironment play a critical role in cancer progression by remodelling the extracellular matrix, promoting angiogenesis, and also supporting tumour cell survival and proliferation. Downregulation of EFNA4 in these cells can lead to a more aggressive tumour phenotype owing to altered cellular functions.

It is noteworthy that GSEA revealed EFNA4 high and ETS1 low group was associated with “EPIDERMAL CELL DIFFERENTIATION”, and consistent with the GSEA analysis results between groups according to the EFNA4 and ETS1 expression levels separately. This further confirmed the opposing effects of EFNA4 and ETS1 on GC. Additionally, the performed GO and KEGG analyses of the DEGs between the EFNA4-high/ETS1-low and EFNA4-low/ETS1-high groups showed a significant enrichment in the cell activation pathway, which harboured 18 subpathways related to immune cell activation, response, and differentiation et al. These results emphasise the roles of EFNA4 and ETS1 in tumour immune cells. Complexity of the immune microenvironment in patients with different clusters of GC using CIBERSORT and ssGSEA analyses.

ETS1 is a transcription factor that is often associated with immune suppression and cancer cell proliferation, whereas EFNA4 may play different roles in immune regulation and intercellular interactions. Therefore, Cluster 2, which was characterised by high ETS1 and low EFNA4 expression, showed an increase in cell subpopulations related to immune suppression, such as naïve B cells, memory B cells, resting dendritic cells, and resting mast cells, which are typically associated with a weaker immune response and may facilitate tumour immune evasion. Previous studies have indicated that the gene copy numbers in gastrointestinal tract marginal zone B-cell lymphomas show an increase in ETS1 and a few surrounding genes in the more aggressive large-cell versions of these tumours^52^. Conversely, Cluster 1, with low ETS1 and high EFNA4 expression, exhibited a more active immune response, with higher proportions of activated NK cells, M0 macrophages, and activated mast cells. These cell types are usually associated with robust immune surveillance and the ability to attack tumour cells. Previous studies have demonstrated that NK cells possess a unique capability to rapidly eliminate nearby cells that exhibit cancer-related surface markers^53^. M0 macrophages are known to produce TNF-α, a cytokine essential for promoting antitumour responses^54^. CD56 bright NK cells, recognised for their high immune activity and crucial role in immune regulation by producing substantial levels of cytokines like IFN-γ and enhancing resistance to ROS-induced cell apoptosis, were markedly prevalent in Cluster 1, suggesting a potent immune response against the tumour^55^. By identifying the immune cells and pathways associated with different GC clusters, this study provides clues for the development of new therapeutic strategies. For example, targeting immune cell types and pathways with low ETS1 and high EFNA4 expression may improve the prognosis of patients with GC.

Tumour purity is a unique feature of GC that affects patient outcomes^56^. Low tumour purity has been linked to reduced survival, quicker relapse, and mesenchymal, invasive, and metastatic characteristics^57–59^. Our study has revealed that cluster 1 exhibited higher tumour purity than cluster 2. High tumour purity has been linked to greater sensitivity to chemotherapy, as activation of proliferation and metabolic pathways leads to increased susceptibility to drugs such as paclitaxel and fluorouracil^60^. EFNA4 is involved in various tumour signalling pathways, whereas ETS1 is linked to tumour immunity and is positively correlated with fibroblast-related genes, contributing to chemotherapy resistance in low-purity tumours. Moreover, the differential expression patterns of EFNA4 and ETS1 and their association with immune checkpoints, such as PDCD1 and TIGIT, revealed their opposing effects in GC. Our analysis has revealed a positive correlation between ETS1 and the immune checkpoints PDCD1 and TIGIT, which are linked to immune dysfunction and tumour progression^61^. The concurrent expression of TIGIT and PD-1 has been associated with immune evasion in GC^62^. In contrast, EFNA4 negatively correlated with these immune checkpoints, further highlighting its potential inhibitory role in tumour progression and immune evasion. Memory T cells and their subsets are important immune markers for evaluating disease stage. The composition of these subsets may reflect the balance between immune suppression and the response to tumour-associated antigens. Wherry et al. in their study suggested that central memory T cells (Tcm) and effector memory T cells (Tem) represent a continuum in a linear differentiation pathway, rather than distinct subsets such as naïve-effector-Tem-Tcm^63^. ETS1 positively influences CD8 + T cell populations, including activated central and effector memory CD8 + T cells, suggesting its importance in the regulation of tumour immunity. EFNA4, in contrast, had no correlation with activated and central memory CD8 + T cells, and negatively affected effector memory CD8 + T cells. This indicates that EFNA4 may play a role in the early stages of GC, as opposed to ETS1. This observation aligns with previous prognostic analyses that demonstrated that increased EFNA4 expression is linked to a more favourable prognosis. Our findings suggest that patients with low ETS1 and high EFNA4 expression levels may experience an improved response in regard to chemotherapy.

scRNA-seq with a high resolution of individual cells provides a better understanding of intratumoural and intertumoral heterogeneities. Given the significant negative correlation between the roles of EFNA4 and ETS1 in immune cells, we developed a useful prognostic model for GC based on the immune cell marker genes EFNA4 and ETS1. However, single-cell sequencing data analysis indicated that the capture of immune cells in gastric tumour tissue is low, which means that relying on the marker genes of immune cells, EFNA4, and ETS1 to construct prognostic models will result in significant errors when evaluating the prognosis of patients with GC. Tumour and endothelial cell clusters comprised a relatively large proportion of the total number of cells captured in EGC and AGC tissues, accompanied by changes in ETS1 and EFNA4 in EGC and AGC. More importantly, the number of tumour cell sub-clusters was changed in AGC compared to that in EGC, and there seems to be some correlation between ETS1 and changes in tumour cells. Prognostic models for GC have been developed and tested based on the marker genes of tumour cell clusters or endothelial cells, EFNA4 and ETS1, and the former can better evaluate the prognosis of patients with GC. It should be noted that we employed tumour cell marker genes for tumour cell cluster identification and did not use the “inferCNV” algorithm for further validation^64,65^. This cluster demonstrates the highly specific expression of genes, such as CLDN4 and CEACAM6, which have been well documented in previous studies as being closely associated with tumourigenesis and progression. Collectively, these findings substantiate both the robustness and accuracy of our tumour cell annotation system. We will incorporate the “inferCNV” algorithm in future analyses to further enhance annotation precision. Additional prospective experiments should be conducted to confirm these findings.

Effective markers for EGC diagnosis and prognostic indicators are of great significance for early screening and clinical treatment of GC. EFNA4 is highly expressed in early gastric cancer, whereas ETS1 expression is low. During the progression of GC, ETS1 tends to be highly expressed to inhibit EFNA4 expression, leading to high ETS1 expression and low EFNA4 expression during the late stages of GC. The differential expression of these two factors at different stages of GC enables them to become effective detection markers for EGC diagnosis, prognosis, and development stages of GC, thereby providing assistance for the clinical prevention and treatment of GC. In addition, owing to the heterogeneity of tumour cells, there is a lack of good biomarker data, and the universality and clinical practicality of existing biomarker detection methods in different populations are not high. In our study, single-cell sequencing results were used to study the use of tumour cell markers with a high proportion in cancer tissues, as well as EFNA4 and ETS1, in order to construct a prognostic model that can better predict the prognosis of patients with GC. These results provide insights for the development of effective clinical prognostic models for GC.

## Conclusion

Our study has elucidated the distinct roles of EFNA4 and ETS1 in GC, as well as their expression patterns in tumour cells at the single-cell level. ETS1 regulates the expression of EFNA4, and the functions of ETS1 and EFNA4 are opposite in GC. These two factors play important roles in the progression of GC and are involved in the cellular processes of epidermal cell differentiation and tumour immune response, as proven through transcriptome and scRNA-seq data of GC. Based on the expression patterns of EFNA4 and ETS1 in GC, a prognostic prediction model was constructed and verified using the TCGA-STAD data. This prognostic prediction model enables refined stratification of gastric cancer patients, thereby guiding personalized therapeutic decision-making and providing a translational framework for optimizing treatment strategies. These findings contribute to a more comprehensive understanding of GC pathogenesis and suggest promising avenues for future research and treatment strategies.

## Supplementary Information

Below is the link to the electronic supplementary material.

Supplementary Material 1

Supplementary Material 2

Supplementary Material 3

Supplementary Material 4

Supplementary Material 5

Supplementary Material 6

Supplementary Material 7

Supplementary Material 8

## Data Availability

All data presented in this study can be obtained from the corresponding author upon reasonable request. Publicly available data employed in this study can be found in TCGA and GEO databases.
