# Supplementary material for: A prognostic risk prediction model for gastric cancer based on the EFNA4 and ETS1 regulatory axis in tumor cells
Source: Sci Rep. 2025 Oct 29;15:37871. doi: 10.1038/s41598-025-21728-6 (PMC12572136; doi:10.1038/s41598-025-21728-6)
Supplement: Supplementary file 4 — Supplementary Material 4 [file 41598_2025_21728_MOESM4_ESM.docx]

**Supplementary Figure**


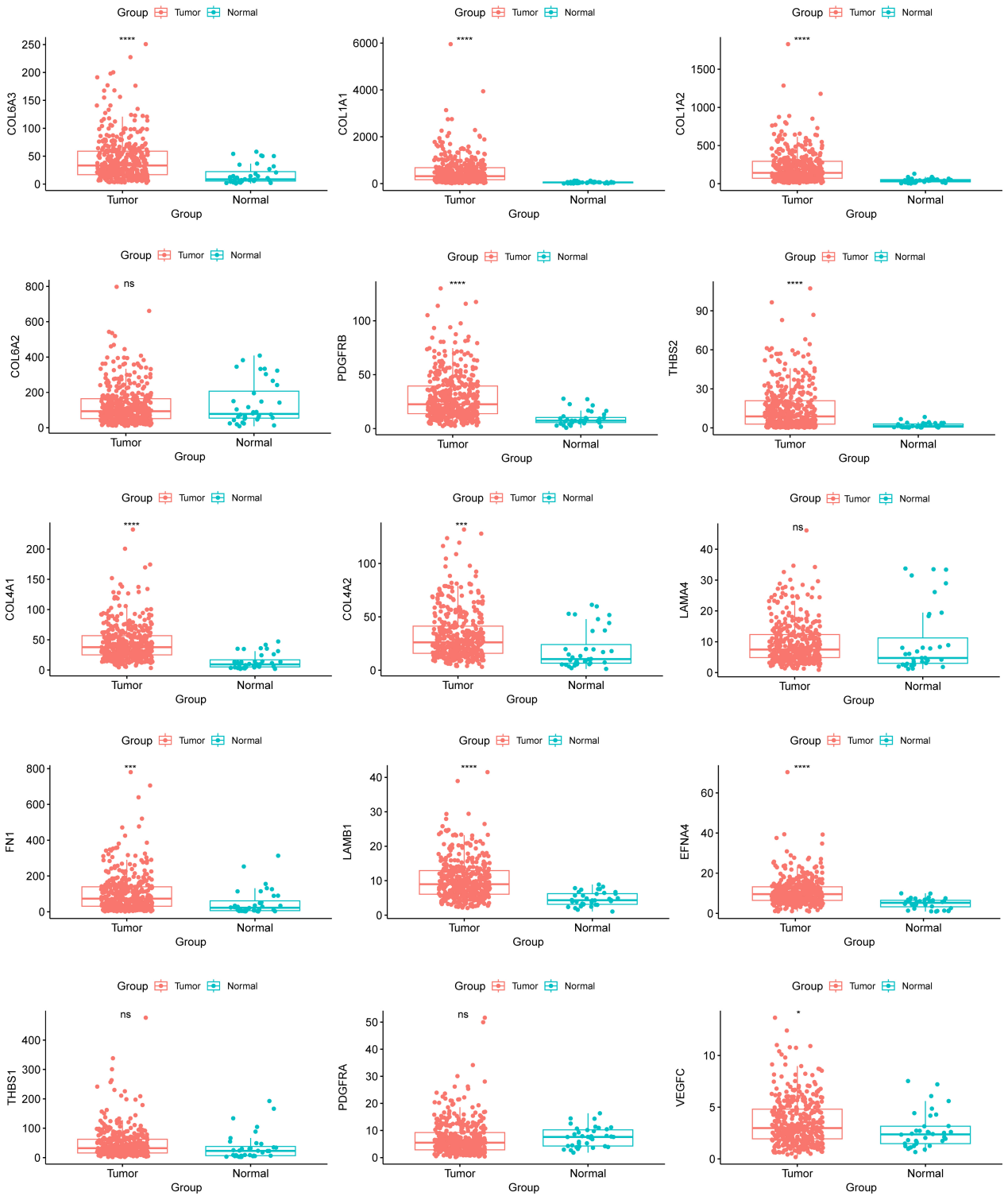


**Supplementary Figure 1. The mRNA expression of survival-related genes (*COL6A3*, *COL1A1*, *COL1A2*, *COL6A2*, *PDGFRB*, *THBS2*, *COL4A1*, *COL4A2*, *LAMA4,* *FN1*, *LAMB1*, *EFNA4*, *THBS1, VEGFC* and *PDGFRA*) in gastric cancer and normal tissues from TCGA data.**


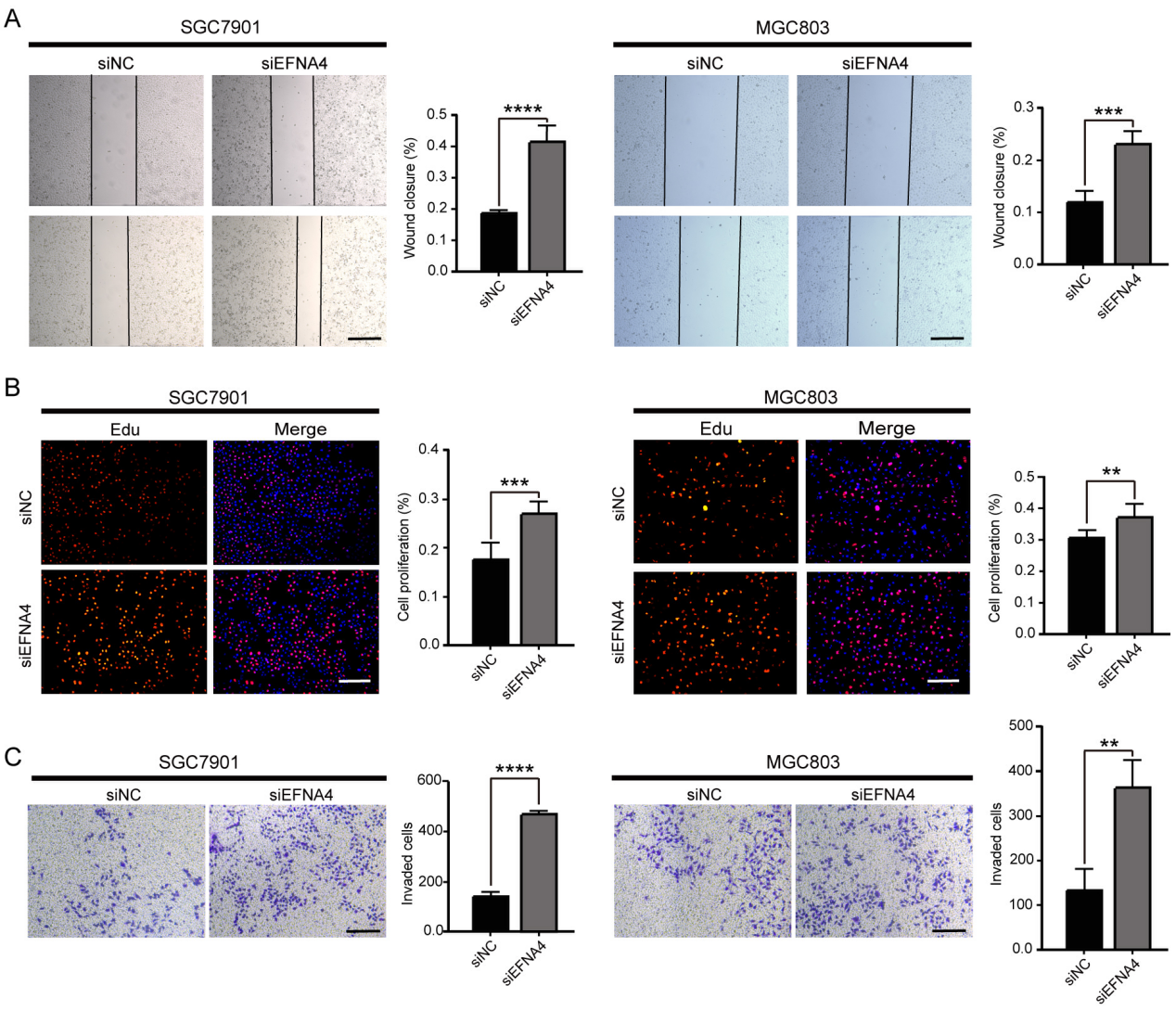


**Supplementary Figure 2. Knockdown of *EFNA4* promotes the proliferation, migration, and invasion of gastric cancer cells**. (a) Wound healing in SGC7901 and MGC803 cells after knockdown of *EFNA4*. (b) EdU assays of cell proliferation after transfection with EFNA4 siRNA. (c) Transwell migration assays in SGC7901 and MGC803 cells after EFNA4 siRNA transfection. Data are presented as the mean ± SEM from three independent experiments. Scale bar: 50 µm. Unpaired two-tailed t test was used for statistical analysis between two groups. **p*-value < 0.05, ***p*-value < 0.01, and ****p*-value < 0.001, *****p*-value < 0.0001.


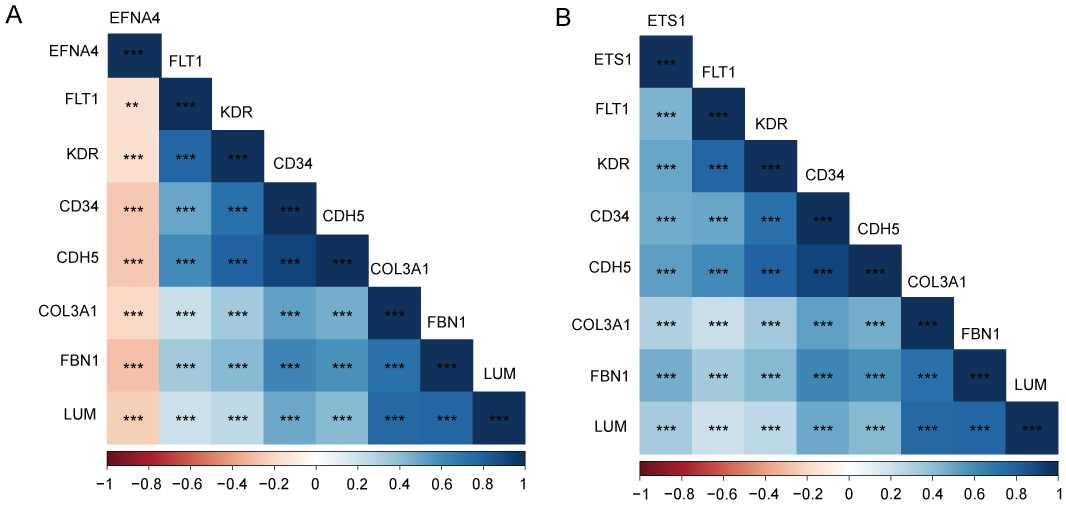


**Supplementary Figure 3. The correlation between the expression levels of target genes with a panel of vascular and fibroblast-related genes.** (A) The co-expression pattern of EFNA4 with vascular and fibroblast genes. (B) ETS1 co-expression with vascular and fibroblast-associated genes. The color intensity indicates correlation strength, and asterisks denote statistical significance.

**
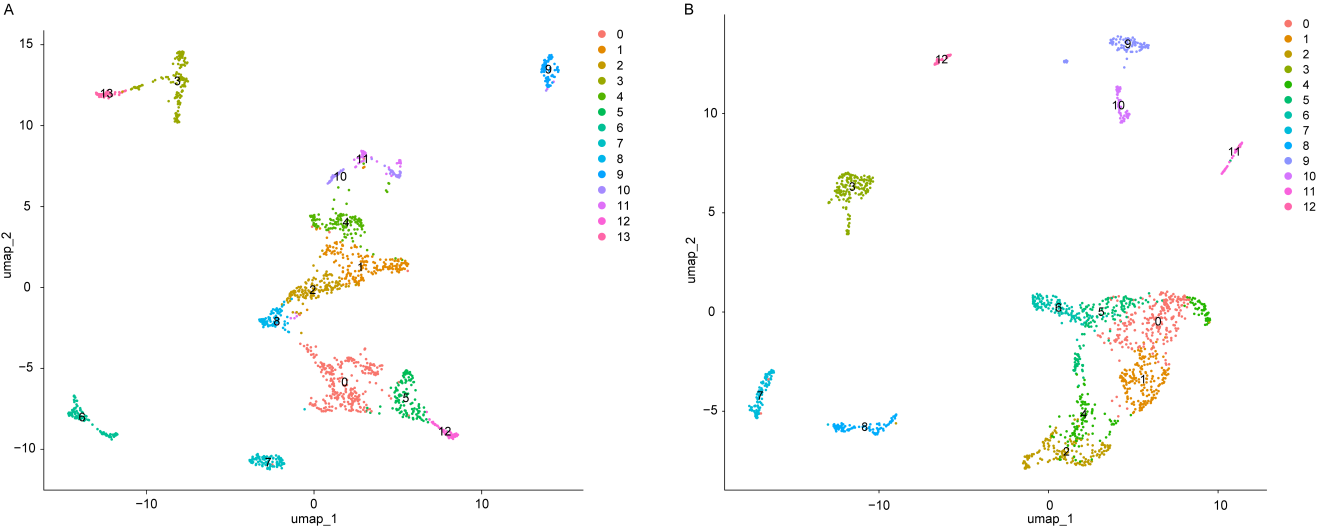
**

**Supplementary Figure 4. The UMAP plot showed the distribution of different clusters of EGC (A) and AGC (B).**


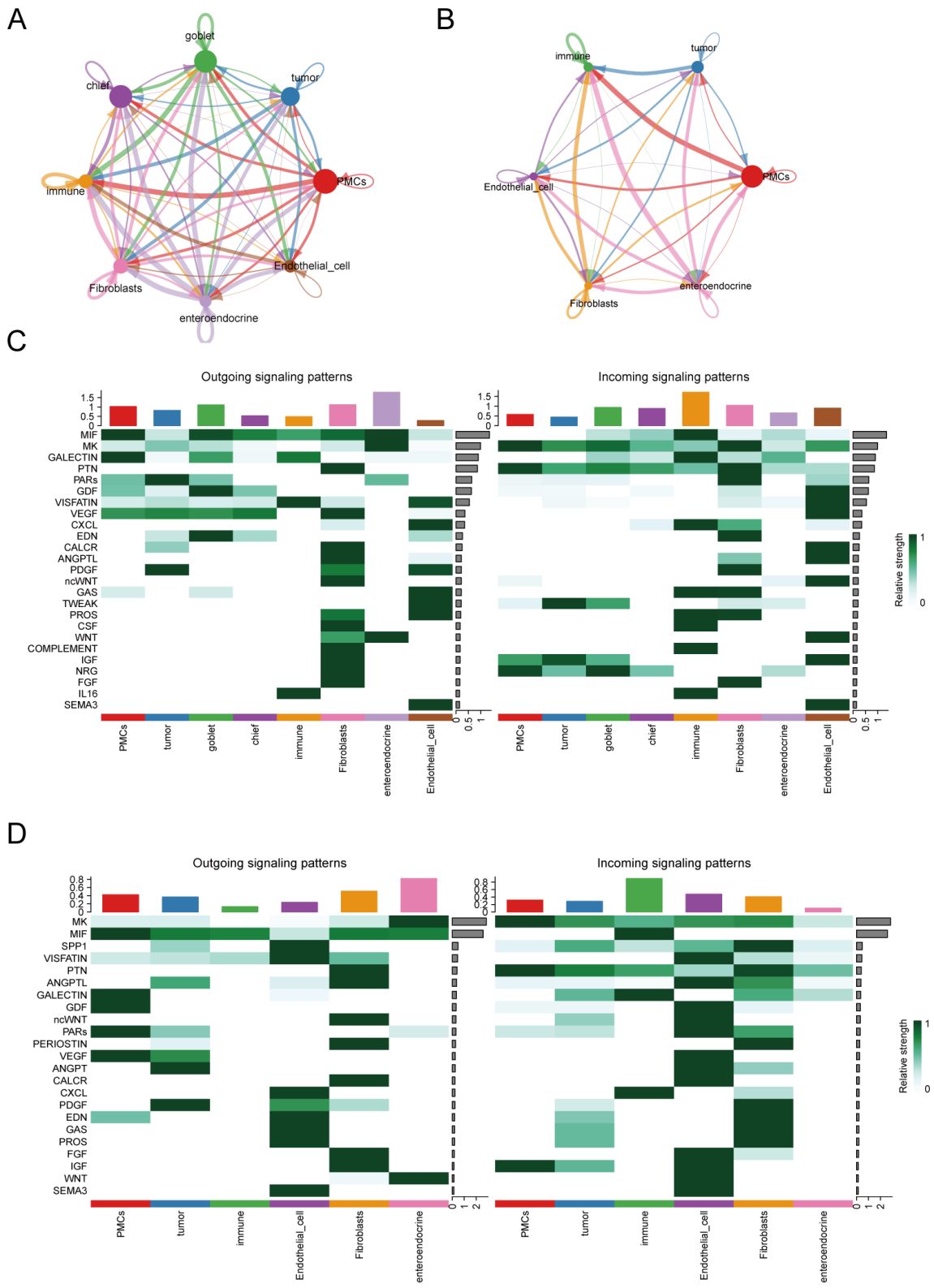


**Supplementary Figure 5. Cellular communication networks and signaling dynamics in gastric cancer progression.** (A) The network of intercellular communication among various cell types within the microenvironment of early gastric cancer (EGC) and (B) advanced gastric cancer (AGC). The nodes and color symbolize distinct cell populations, the connecting lines illustrate the interactive dynamics, and the thickness of the connecting line denotes the diversity or intensity of these interactions. The signaling landscape in (C) EGC and (D) AGC, and the left chart for "Outgoing signaling patterns," highlights the spectrum of signal molecules secreted by different cells, and the right chart for "Incoming signaling patterns," showcasing the array of signals received by the cell populations. The color of bars indicates the strength of the intensity of signaling.





**Supplementary Figure 6. Predictive modeling and survival analysis in endothelial cell-mediated tumor progression.** (A) The trajectories of coefficients for various endothelial cell marker genes in a lasso regression model. (B) The selection of the optimal lambda parameter in the lasso model through cross-validation. (C) The hazard ratios for endothelial cell marker genes by a multivariate Cox proportional hazards regression analysis. (D) Kaplan-Meier curves depicts survival probabilities over time for patients in low- and high-risk groups. (E) ROC curves at 1, 3, and 5 years assesses the time-specific predictive accuracy of the survival model in TCGA dataset. (F) The multivariate Cox regression analysis evaluates the clinical characteristics and risk scores on survival.
