# Supplementary figures and images for: A prognostic risk prediction model for gastric cancer based on the EFNA4 and ETS1 regulatory axis in tumor cells
Source: Sci Rep. 2025 Oct 29;15:37871. doi: 10.1038/s41598-025-21728-6 (PMC12572136; doi:10.1038/s41598-025-21728-6)

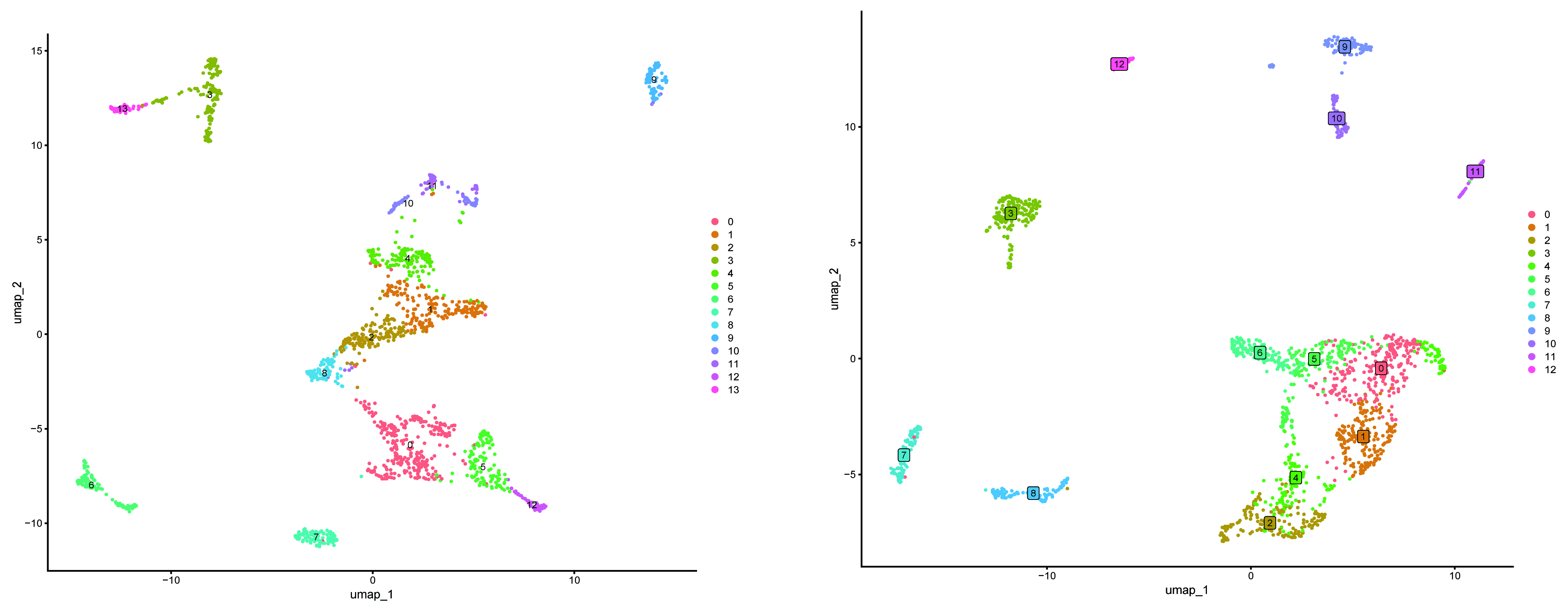

Supplement: Supplementary file 6 — Supplementary Material 6 [file 41598_2025_21728_MOESM6_ESM.tif]
